# Frequent and strong cold‐air pooling drives temperate forest composition

**DOI:** 10.1002/ece3.11126

**Published:** 2024-04-01

**Authors:** Melissa A. Pastore, Aimée T. Classen, Anthony W. D'Amato, Marie E. English, Karin Rand, Jane R. Foster, E. Carol Adair

**Affiliations:** ^1^ Rubenstein School of Environment and Natural Resources University of Vermont Burlington Vermont USA; ^2^ Gund Institute for Environment, University of Vermont Burlington Vermont USA; ^3^ USDA Forest Service, Northern Research Station St. Paul Minnesota USA; ^4^ Ecology and Evolutionary Biology Department University of Michigan Ann Arbor Michigan USA; ^5^ University of Michigan Biological Station Pellston Michigan USA; ^6^ USDA Forest Service, Southern Research Station Knoxville Tennessee USA

**Keywords:** cold‐air drainage, cold‐air pooling, complex topography, forest composition, microclimate, microrefugia, mountains, temperature inversions

## Abstract

Cold‐air pooling is an important topoclimatic process that creates temperature inversions with the coldest air at the lowest elevations. Incomplete understanding of sub‐canopy spatiotemporal cold‐air pooling dynamics and associated ecological impacts hinders predictions and conservation actions related to climate change and cold‐dependent species and functions. To determine if and how cold‐air pooling influences forest composition, we characterized the frequency, strength, and temporal dynamics of cold‐air pooling in the sub‐canopy at local to regional scales in New England, USA. We established a network of 48 plots along elevational transects and continuously measured sub‐canopy air temperatures for 6–10 months (depending on site). We then estimated overstory and understory community temperature preferences by surveying tree composition in each plot and combining these data with known species temperature preferences. We found that cold‐air pooling was frequent (19–43% seasonal occurrences) and that sites with the most frequent inversions displayed inverted forest composition patterns across slopes with more cold‐adapted species, namely conifers, at low instead of high elevations. We also observed both local and regional variability in cold‐air pooling dynamics, revealing that while cold‐air pooling is common, it is also spatially complex. Our study, which uniquely focused on broad spatial and temporal scales, has revealed some rarely reported cold‐air pooling dynamics. For instance, we discovered frequent and strong temperature inversions that occurred across seasons and in some locations were most frequent during the daytime, likely affecting forest composition. Together, our results show that cold‐air pooling is a fundamental ecological process that requires integration into modeling efforts predicting future forest vegetation patterns under climate change, as well as greater consideration for conservation strategies identifying potential climate refugia for cold‐adapted species.

## Graphical abstract

To determine if and how cold‐air pooling influences forest composition, we surveyed tree composition and characterized the frequency, strength, and temporal dynamics of cold‐air pooling in the sub‐canopy at local to regional scales in New England, USA. We found that cold‐air pooling was frequent (19–43% seasonal occurrences) and that sites with the most frequent inversions displayed inverted forest composition patterns across slopes with more cold‐adapted species, namely conifers, at low instead of high elevations. Cold‐air pooling occurred across seasons and times of day, and, in some locations, was surprisingly more frequent during the daytime than the nighttime.

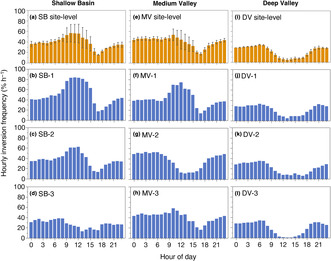

## 1 INTRODUCTION

Ecosystems and communities are shaped by many interacting physical, chemical, and biological processes, but the phenomenon of cold‐air pooling is frequently overlooked. Cold‐air pooling is a topoclimatic process that can create inverted and nonlinear temperature gradients across elevation in areas with sheltered topographic features, such as sinkholes, depressions, gullies, basins, and hill or mountain valleys (Clements et al., 2003; Geiger, 1965; Lundquist & Cayan, 2007; Mahrt et al., 2001; Whiteman et al., 2001). Cold‐air pools primarily form around sunset when upslope radiative surface cooling leads to cold, downslope airflow (katabatic winds) or, less commonly, from in situ radiative surface cooling in valley bottoms (Kelsey et al., 2019; Kiefer & Zhong, 2013; Rupp et al., 2020). Cold, dense air settles in low‐lying areas and shifts them from being some of the warmest spots on the landscape to the coldest (Clements et al., 2003). Cold‐air pooling occurs worldwide (Pastore et al., 2022) and affects other key environmental conditions, including soil temperature and moisture, vapor pressure deficit, frost frequency, and snowpack depth and duration (Curtis et al., 2014; Daly et al., 2010; Maclean et al., 2017; Novick et al., 2016; Pepin et al., 2011; Reeves & Stensrud, 2009). Generally considered nocturnal wintertime phenomena that dissipate soon after sunrise (Reeves & Stensrud, 2009; Whiteman et al., 1999, 2001; Wolyn & McKee, 1989), cold‐air pools may occur during the daytime and in all seasons (Bigg et al., 2014; Daly et al., 2007; Novick et al., 2016; Pypker, Unsworth, Lamb, et al., 2007; Pypker, Unsworth, Mix, et al., 2007; Rupp et al., 2020). In fact, recent evidence suggests that in some landscapes, such as the Cascade Range in Oregon, USA, cold‐air pooling is the norm rather than the exception (Rupp et al., 2020).

Frequent or strong cold‐air pools could act as microrefugia for species and ecosystem functions as the climate changes (Dobrowski, 2011; McLaughlin et al., 2017; Morelli et al., 2016; Pastore et al., 2022; Patsiou et al., 2017). Due to a lack of vertical mixing, cold‐air pools become partially or fully decoupled from the overlying free atmosphere (Yao & Zhong, 2009). Consequently, cold‐air pooling areas may be buffered and/or decoupled from regional warming (Daly et al., 2010). Thus, the failure of coarse‐grained climate models (typically >50 km) to simulate this fine‐scale process may hinder the accuracy of climate change, species distribution, and ecosystem function predictions (Daly, 2006; Daly et al., 2010; Fridley, 2009; Lenoir et al., 2017; Maclean et al., 2017; Nadeau et al., 2017, 2022; Potter et al., 2013) and limit the effectiveness of conservation actions focused on sustaining cold‐dependent species and functions.

Cold‐air pools can act as environmental filters that selectively favor and exclude certain species. For instance, the low temperatures and frost events characteristic of cold‐air pools may reduce the productivity, survival, and regeneration of many broadleaved species, releasing cold‐adapted species such as conifers from competitive pressure (Pastore et al., 2022). Isolated studies indicate that these buffered pockets of cooler, moister conditions harbor high‐elevation species where low‐elevation species are predicted to occur (Ai‐liang, 1981; Daubenmire, 1980; Finocchiaro et al., 2023; Millar et al., 2018; Westergren et al., 2018). For example, in the Great Basin of the western United States, the upland conifer limber pine (*Pinus flexilis*) is disproportionately found in low‐elevation ravine areas that experience cold‐air pooling (Millar et al., 2018). Cold‐adapted species that migrate upslope in response to warming may ultimately become locally extinct as their suitable habitat shrinks (Urban, 2018). However, those that inhabit or migrate into buffered low‐elevation areas prone to cold‐air pooling may persist locally for longer periods, which can facilitate larger scale range shifts and gene transfers that enhance adaptation (Hannah et al., 2014; Morelli et al., 2016). Additionally, these effects of cold‐air pooling microclimates on forest composition may feed back to impact the abiotic environment. For example, coniferous species can gradually modify soil conditions and light availability in ways that further exclude broadleaved species. These coniferous forest communities can acidify soils, maintain year‐round shade, preserve snowpack, retain soil moisture, and promote soil carbon accrual and storage (Boča et al., 2014; Jevon et al., 2019; Pastore et al., 2022; Siccama, 1974). Furthermore, valley floors and topographic depressions where cold‐air pooling occurs may accumulate greater amounts of soil resources, influencing community composition (Frei et al., 2023) and potentially reinforcing effects of plant communities on ecosystem functions like water storage. Thus, through such climate–plant–soil feedbacks, cold‐air pools may not only preserve species but also critical ecosystem functions that are vulnerable to regional warming (Pastore et al., 2022).

Cold‐air pooling likely shapes the composition and function of ecosystems, and, indeed, was hypothesized to explain vegetational inversions in the mid‐20th century (Grigal, 1968; Linn, 1957; Loucks, 1962; White, 1976). However, relatively few studies have empirically linked cold‐air pooling frequency and intensity to patterns of forest composition. Rather, most cold‐air pooling studies focus on the air above forest canopies and in unforested areas (De Frenne & Verheyen, 2016). However, isolated studies suggest that above‐ versus below‐canopy cold‐air pooling dynamics differ (Kelsey et al., 2019; Pypker, Unsworth, Lamb, et al., 2007). This is particularly important because key biotic–abiotic relationships and drivers of future forest dynamics, namely tree regeneration, occur beneath the forest canopy and respond to the near‐ground microenvironment. Moreover, previous studies generally focused on inversion formation and dissipation during single cold‐air pooling events at single locations. To understand the regional effects of cold‐air pooling on forest composition, we need to explore the frequency and strength of cold‐air pooling over broad temporal and spatial scales.

**FIGURE 1:**
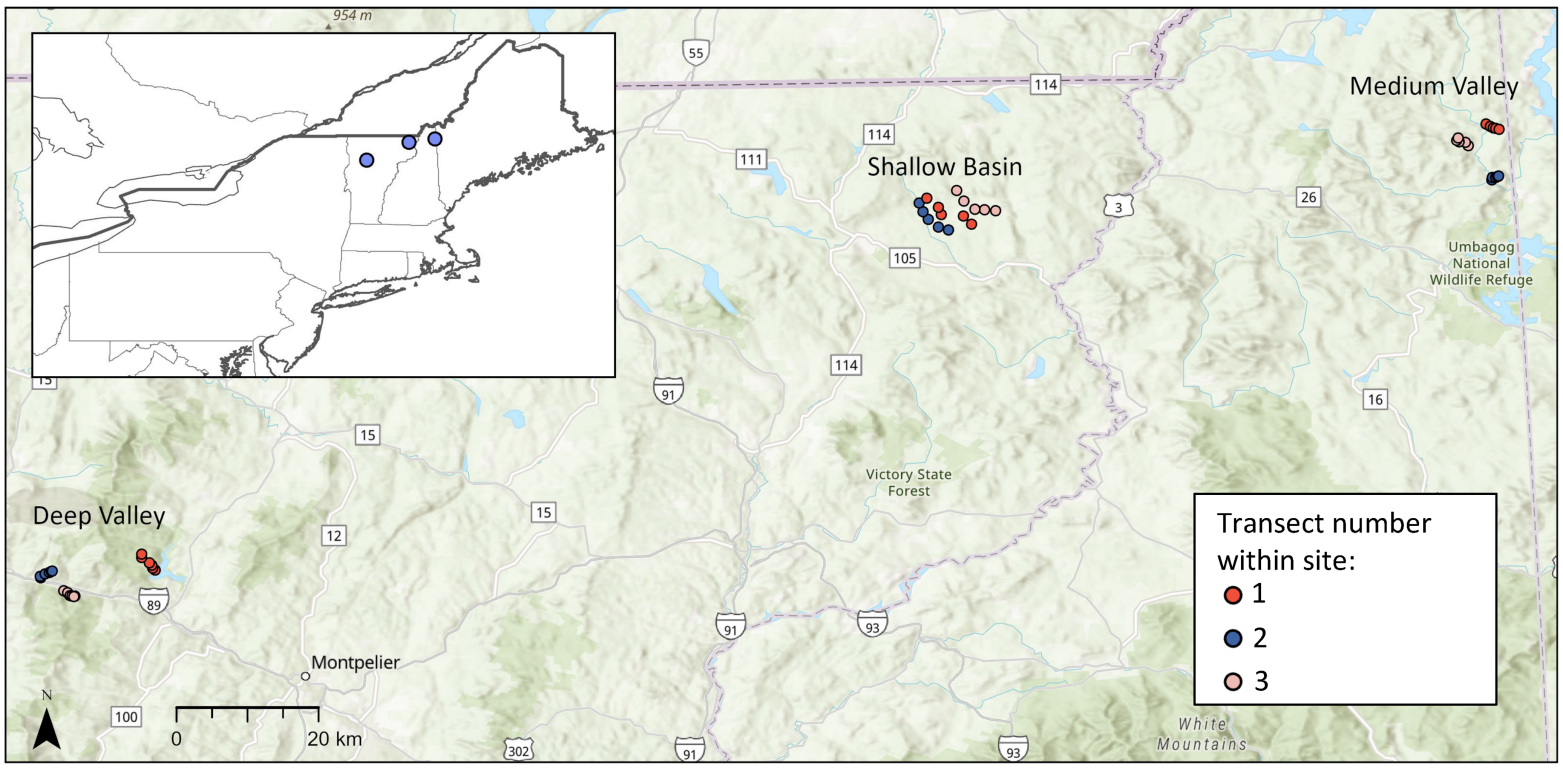
Map of plot locations across 9 transects in 3 sites (Deep Valley, Shallow Basin, Medium Valley).

The northeastern United States provides a natural experiment to explore cold‐air pooling dynamics and assess influences on forest composition because this region contains diverse topography and encompasses the temperate‐boreal ecotone, where warm‐ and cold‐adapted species exist in close proximity and are distributed along climatic gradients (Cogbill & White, 1991; Kupfer & Cairns, 1996; Siccama, 1974). Additionally, the northeastern United States is one of the fastest warming regions in the world (Karmalkar & Bradley, 2017; Wuebbles et al., 2017) and is a globally important carbon sink (Dubayah et al., 2022; Jiang et al., 2022). Thus, we sought to determine if and how cold‐air pooling influences forest composition by establishing a network of elevational transects across three sites that represent distinct topographies of the northeastern USA (Figure 1). We continuously measured air temperatures across these transects to characterize the frequency, strength, and temporal dynamics of cold‐air pooling in the forest sub‐canopy at local to regional scales and we assessed plant community composition and temperature preferences expressed as the community temperature index (CTI; Devictor et al., 2012). We hypothesized that cold‐air pooling would affect forest composition by favoring cold‐preference tree species at low elevations, leading to inverted forest composition patterns with elevation (i.e., decreasing CTI as elevation decreases). Specifically, we predicted that in sites with frequent cold‐air pooling, cold‐preference species (i.e., conifers like *Picea rubens* and *Abies balsamea*) would dominate at low elevations and transition to warm‐preference species (i.e., temperate hardwoods like *Acer saccharum* and *Fagus grandifolia*) along upper slopes.

## 2 MATERIALS AND METHODS

### 2.1 Study sites

To characterize cold‐air pooling dynamics and investigate links to forest composition, we deployed temperature sensors and surveyed forest composition across nine elevational transects spanning three sites in New England, USA (Figure 1). We confirmed a priori that cold‐air pooling events occur at coarse watershed scales in each region via 1‐km gridded Land Surface Temperature data from MODIS satellites. We chose three sites with distinct topographies typical of the northeastern region, including a shallow basin (hereafter ‘Shallow Basin’, SB), a mountain‐valley landscape with medium relief (hereafter ‘Medium Valley’, MV), and a mountain‐valley landscape with deep relief (hereafter ‘Deep Valley’, DV) (Table 1, Figure 1). The Shallow Basin site is located in the Nulhegan Basin portion of the Silvio O. Conte National Fish and Wildlife Refuge in the Northeast Kingdom of Vermont (44.846635°, −71.75817°) and is a ~16‐mile wide crater‐like basin surrounded by hills with a flat to gently rising interior; mean annual temperature (MAT) is 5.0°C and mean annual precipitation (MAP) is 1225 mm (Daymet data, Thornton et al., 2020, Table 1, Figure 1). The Medium Valley site is in the Dartmouth College Second College Grant in northern New Hampshire along the Maine border (44.925853°, −71.08554°) and is a mountain‐valley landscape with medium relief for the region; MAT is 4.1°C and MAP is 1247 mm (Daymet data, Thornton et al., 2020, Table 1, Figure 1). The Deep Valley site is in Vermont's northern Green Mountains (44.384743°, −72.85045°) and is a mountain‐valley landscape with deep relief that includes some of the tallest peaks in the state; MAT is 6.7°C and MAP is 1323 mm (Daymet data, Thornton et al., 2020, Table 1, Figure 1).

**TABLE 1:** Site‐ and transect‐level characteristics.

Site characteristics	Shallow Basin	Medium Valley	Deep Valley
Forest type	Northern hardwood to spruce‐fir transition zone	Northern hardwood to spruce‐fir transition zone	Northern hardwood
Topography	Lowland basin surrounded by hills and some mountainous uplands	Mountain‐valley	Mountain‐valley
Land‐use history	Extensive forest harvesting 1960–1980; protection as refuge beginning in 1999	Low intensity forest harvesting in 1940s and 1960s	Clearing for agriculture followed by subsequent abandonment in 19th century; low intensity harvesting in early 1900s
Mean annual temperature^a^	5.0°C	4.1°C	6.7°C
Mean annual precipitation^a^	1225 mm	1247 mm	1323 mm
Number of plots	15	15	18

Transect characteristics	SB‐1	SB‐2	SB‐3	MV‐1	MV‐2	MV‐3	DV‐1	DV‐2	DV‐3
Latitude (degrees)	44.84594	44.83812	44.85584	44.95314	44.88953	44.93489	44.40261	44.38897	44.36265
Longitude (degrees)	−71.76227	−71.78370	−71.72853	−71.07336	−71.07037	−71.11289	−72.77440	−72.90343	−72.87351
Elevation range (m a.s.l.)	369–492	390–442	392–473	423–605	413–585	459–639	238–547	191–510	151–458
Mean northness^b^	−0.04	0.16	0.16	0.26	−0.52	−0.74	−0.78	−0.60	0.61
Mean eastness^c^	−0.11	−0.12	−0.36	−0.52	−0.69	0.07	−0.20	−0.67	0.26
Mean slope (degrees)	7	5.2	4	7.8	12.6	11	15	23	20.5
Period of sensor measurements (m/d/y)	9/25/21–3/12/22	9/25/21–3/12/22	9/25/21–3/12/22	8/21/21–6/30/22	8/21/21–6/30/22	8/21/21–6/30/22	8/21/21–6/7/22	8/1/21–6/7/22	8/1/21–6/7/22

^a^ Daymet data from 2000 to 2022 (Thornton et al., 2020).

^b^ Northness = cosine(aspect), calculated with aspect in radians; northness ranges from −1 at 180° (south) to 1 at 0° (north).

^c^ Eastness = sine(aspect), calculated with aspect in radians; eastness ranges from −1 at 270° (west) to 1 at 90° (east).

We established three transects within a 75 km^2^ area in each site during the mid to late growing season of 2021 (Figure 1). We placed transects in representative vegetation for each site and avoided recent disturbances, such as harvesting, based on satellite imagery and local expertise (Table 1). Each transect contained five to six circular plots (0.04 ha each) across an elevation gradient, with the lowest plot positioned within 0.25–0.5 km of the bottom of the valley or basin (i.e., bottom of the cold‐air pool) and the highest plot positioned near the top (within about 1 km). Relief differed across sites such that the elevation difference from the top to the bottom of transects was, on average, 85 m at Shallow Basin, 178 m at Medium Valley, and 312 m at Deep Valley (Table 1). The maximum depth of cold‐air pools is dependent on local mountain height and reservoir shape (Kelsey et al., 2019), and thus we designed our transects with the aim of meeting or exceeding fine‐scale cold‐air pool height. Because of the unique crater‐like topography of the Shallow Basin site, we spaced plots along transects from the interior of the basin outward and generally upslope. In total, we established 48 plots in nine transects across the three sites (Figure 1). Transects are numbered 1 through 3 within each site (Table 1, Figure 1). Based on Daymet data (Thornton et al., 2020) for each site from years 2000 to 2022, mean annual temperature was at the warmer end of the range in both 2021 and 2022 and precipitation was at the lower end of the range during 2021 and closer to average in 2022.

### 2.2 Sensor deployment and data collection

In each plot, we measured elevation, latitude, longitude (Garmin GPSMAP 64st), slope, and aspect. We measured sub‐canopy air temperatures in each plot with Thermochron iButton temperature sensors and dataloggers (DS1922L, Maxim Integrated Products, Inc., Sunnyvale, CA, USA) shielded from solar radiation and precipitation with open‐base plastic containers. Two sensors per plot were hung from separate, randomly selected trees 1.5 m above the ground surface and logged temperature in 1‐h intervals over the following periods: Shallow Basin: September 25, 2021—March 12, 2022; Medium Valley: August 21, 2023—June 30, 2022; Deep Valley: August 21, 2021—June 7, 2022 at DV‐1 and August 1, 2021—June 7, 2022 at DV‐2 and DV‐3 (Table 1; all times in local Eastern Standard Time). Within each plot, we recorded the species of all living overstory trees with diameter at breast height (DBH) ≥ 10.16 cm at the time of plot establishment and, within a central, circular subplot (0.01 ha), the species of all living understory trees with DBH < 10.16 cm and height > 30.5 cm.

To explore the influence of edaphic properties on forest composition across elevation gradients, we measured soil depth, gravimetric soil water content, soil pH, and soil nitrogen. We determined soil depth (organic + mineral) by inserting a metal probe 1.8 m from plot center at 60, 180, and 360° azimuths until reaching a restrictive layer within the top 90 cm and then averaged the three measurements per plot. We collected 8 mineral soil cores (0–10 cm) per plot starting beneath the organic layer using a soil corer (AMS mini soil probe; 2 cm diameter; AMS, Inc.). We combined the cores in a plastic bag, transported them to the lab on ice, and then homogenized the soil within each plot by sieving (mesh size 2 mm). We measured soil pH using a 10 g wet soil subsample (Mettler Toledo S220 SevenCompact benchtop pH/ion meter in supernatant with soil:0.01 M CaCl_2_ ratio of 1:3; Mettler Toledo, LLC). We determined gravimetric soil water content by drying a 10 g wet soil subsample at 60°C for at least 48 h until a constant mass was reached (adapted from Topp & Ferré, 2002). Samples were then ground (BioSpec Mini‐BeadBeater‐96) and weighed to 19.95–20.05 mg in tin capsules for analysis of total nitrogen concentrations using an elemental analyzer (Elementar UNICUBE in CN mode, Hanau, Germany) at University of Vermont's George D. Aiken Forestry Sciences Lab.

### 2.3 Calculation of cold‐air pooling characteristics

We used an approach similar to Noad and Bonnaventure (2022) to characterize cold‐air pooling at each transect. First, we determined whether an inversion was present or absent during each single hourly timestep at each transect (when present, this is also known as a ‘point inversion’) via two methods. In the first method, we calculated the surface lapse rate (SLR) as the slope of the least squares linear regression of temperature by elevation across a transect. An inversion is present when the surface lapse rate (slope) is >0°C m^−1^ for a timestep (i.e., inverted surface lapse rate, ISLR) (Noad & Bonnaventure, 2022). Because of the unique topography and low relief at Shallow Basin, where elevation was similar (within the typical margin of error of GPS units) among the first three plots of the SB‐2 and SB‐3 transects, we also used a second method of identifying inversions by comparing temperatures of the plot positioned first (low‐elevation) and last (high‐elevation) in a transect. With this method, an inversion is present when temperature of the last/high plot (*T*_high_) is greater than the temperature of the first/low plot (*T*_low_; *T*_high_ > *T*_low_) (Joly & Richard, 2022).

Inversion frequency is the proportion of a given time period that inversions are present (i.e., the number of point inversions per total number of timesteps per period, as a percentage) (Bourne et al., 2010; Noad & Bonnaventure, 2022). We compared inversion frequencies using both methods of identifying inversions (ISLR vs. *T*_high_ > *T*_low_) and found they were strongly positively correlated with a nearly 1:1 fit (*R* ^2^ = 0.9 and *m* = 0.9 for linear regression of monthly inversion frequencies for each transect calculated via the two methods). Thus, we only show results from the *T*_high_ > *T*_low_ method here, which was also the more conservative method of identifying inversions (i.e., tended to lead to modestly lower inversion frequencies). We calculated frequency by each season (percentage of hourly timesteps with inversion presence per season), month (percentage of hourly timesteps with inversion presence per month), and, to show predominant diurnal patterns, by each hourly time interval (percentage of dates with inversion presence for a given hourly interval). For seasonal patterns, date ranges were selected based on data availability and are as follows: ‘Late summer 2021’: August 21, 2021—September 24, 2021; ‘Fall 2021’: September 25, 2021—December 21, 2021; ‘Winter 2021/22’: December 22, 2021—March 12, 2022; ‘Spring 2022’: March 13, 2022—June 7, 2022. For monthly patterns, all months with complete data for each transect were used. For diurnal patterns, data were restricted to the widest timeframe in common among all transects, which covered the fall and winter seasons (September 25, 2021—March 12, 2022).

For every inversion, we calculated two inversion intensity metrics. First, we determined the temperature gradient during each timestep when an inversion was present, specifically the ISLR in units of °C 100 m^−1^ (hereafter ‘inversion strength’). This metric indicates how much air temperature increases with increasing elevation across a transect during an inversion (Bradley et al., 1992; Noad & Bonnaventure, 2022) and is useful for comparing inversion strength across sites that differ in the total amount of elevation spanned. Second, we calculated the ‘temperature difference’ between the last and first plot of a transect (*T*_high_–*T*_low_) during each timestep when an inversion was present (Joly & Richard, 2022). This metric is not standardized by elevation and thus indicates the temperature difference across an entire transect. We summarized each of these metrics (inversion strength and temperature difference) to calculate means by season, month, and by each hourly time interval, as above. We also determined the maximum temperature difference across each entire transect during each season.

We calculated among‐ versus within‐site variability in seasonal and hourly inversion frequency and strength. Within‐site variability is the standard error of the mean across all 3 transects per site for a given season or hourly interval. Among‐site variability is the standard error of the mean across all 3 sites for a given season or hourly interval.

### 2.4 Calculation of community temperature index (CTI)

The community temperature index (CTI) reflects the relative composition of warm‐ and cold‐preference species in a community (Christiansen et al., 2022; Devictor et al., 2012). We first calculated a preferred temperature for each recorded tree species (Table S1) by averaging the 30‐year (1981–2010) temperature mean from CHELSA 1‐km gridded temperature data (Karger et al., 2017, 2018) across its geographic range in North America (Prasad & Iverson, 2003; Witthoeft, 2021), which is sometimes referred to as a “Species Temperature Index” or “Indicator Value.” The database of geographic ranges contains all species recorded in our plots except for *Betula cordifolia*, which constituted 0.3% of the overstory and 0.2% of the understory trees we recorded, and *Amelanchier* spp., which constituted 0.06% of the overstory and 1.8% of the understory trees we recorded. Thus, for *B. cordifolia* we substituted the temperature preference for *Betula papyrifera*, and for *Amelanchier* spp. we used the mean 30‐year (1981–2010) temperature across its US range, extracted from the predictor statistics of the USDA Forest Service Climate Change Tree Atlas (Peters et al., 2020). When we could not distinguish *Picea mariana* from *P. rubens* where they co‐occur at Shallow Basin, as they readily hybridize, we recorded the species as ‘black/red spruce’ and thus averaged the preferred temperatures of those two species for that designation. For each plot, we calculated CTI separately for the overstory and understory communities as the abundance‐weighted mean (by species proportions using tree counts) of preferred temperature values of all overstory or understory tree species present (Devictor et al., 2012).

To explore which tree functional groups were driving CTI patterns, we divided species based on whether they are coniferous or broadleaved and further divided the broadleaved group into whether their temperature preferences fall within the range of the conifers (“cold‐broadleaved”) or above the range of the conifers (“warm‐broadleaved”) (Table S1). We performed least squares linear regressions of CTI and the abundance of each tree functional group against elevation by site and calculated 95% confidence intervals for each fit.

## 3 RESULTS

### 3.1 Seasonal cold‐air pooling dynamics

**FIGURE 2:**
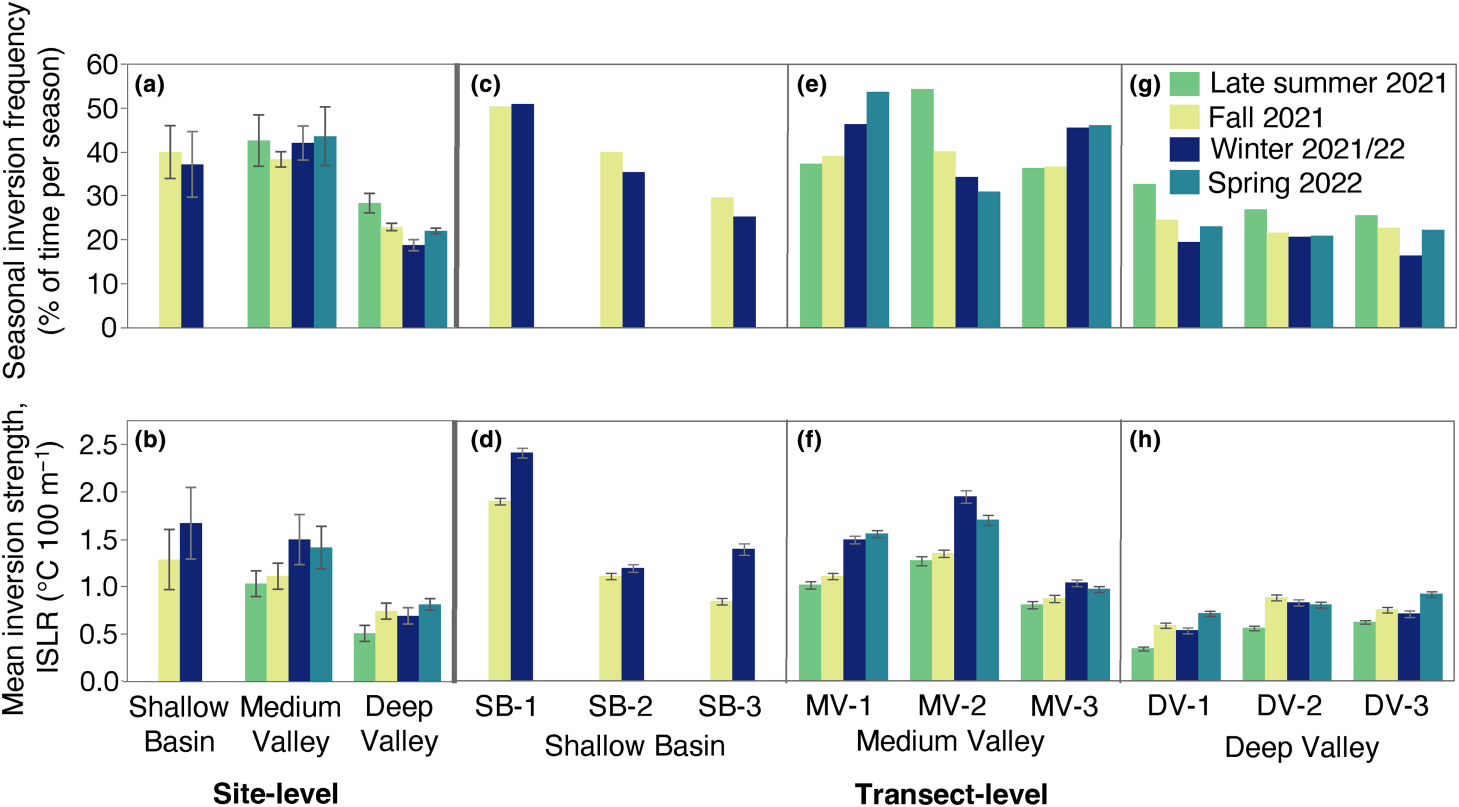
Seasonal cold‐air pooling dynamics at the site‐level (a, b) and transect‐level (c, d: Shallow Basin; e, f: Medium Valley; g, h: Deep Valley). Top row: Seasonal frequency of temperature inversions, calculated as the percentage of hourly timesteps per season that an inversion was present. Bottom row: Mean inversion strength by season, calculated as the mean decline in air temperature with decreasing elevation on a standardized scale across transects (ISLR, units of °C 100 m^−1^). Error bars = ± 1 SE. Each seasonal site‐level bar in a‐b is the mean ± 1 SE of 3 transects. Each seasonal transect‐level bar in (d, f, h) is the mean ± 1 SE across all hourly timesteps when an inversion was present within a given season. Note that data from Shallow Basin are only available from fall and winter.

We found frequent inversions at all three of our sites, with mean frequencies ranging from 19 to 43% of hourly timesteps per season across sites (Figure 2a). At the site‐level, we did not observe differences in inversion frequency across seasons at the Shallow Basin and Medium Valley sites, but at the Deep Valley site, inversion frequency was highest in late summer, when inversions occurred during 28% of timesteps, and lowest in winter, when inversions occurred during 19% of timesteps (Figure 2a, monthly frequencies in Table S2).

Overall, inversions at the Shallow Basin and Medium Valley sites were both more frequent (by 1.5–2.2×, depending on season) and more variable than at the Deep Valley site (Figure 2a,c,e,g, Table S3). Within‐site variability in seasonal inversion frequency in the Medium Valley and Deep Valley sites was substantially lower than among‐site variability (1.2–5.3× lower in Medium Valley and 3.2–17.3× lower in Deep Valley, depending on season, Table S3). In contrast, within‐site variability in seasonal inversion frequency in the Shallow Basin site was 1.1× greater than among‐site variability (Table S3).

We observed strong inversions in all the seasons we measured (Figure 2b–h, Figure S1). Mean inversion strength (ISLR) varied from 0.5–1.7°C 100 m^−1^ depending on site and season and, as with inversion frequency, it was greater at both the Shallow Basin and Medium Valley sites compared to the Deep Valley site in all seasons (Figure 2b,d,f,h, Table S3). However, unlike with inversion frequency, we observed differences in mean strength among seasons at each site (Figure 2b, Table S4). Specifically, inversions were 30% stronger in winter than fall at the Shallow Basin site and 36% stronger in winter and spring than late summer and fall at the Medium Valley site (Figure 2b). At the Deep Valley site, inversions were weaker in late summer than in other seasons (Figure 2b). Transect SB‐1 had the highest seasonal mean inversion strength (ISLR of 2.4°C 100 m^−1^ in winter, Figure 1d) and highest maximum temperature difference reached across a transect (16.2°C, Figure S1d, mean monthly inversion temperature differences in Table S5).

We observed the most within‐site variability in inversion strength in the Shallow Basin site, followed by the Medium Valley site, and the least in the Deep Valley site (Figure 2b,d,f,h, Table S3). Within‐site variability in inversion strength in the Shallow Basin site was substantially greater than among‐site variability (by 98% in fall and 26% in winter), whereas within‐site variability in the Medium Valley site and, in particular, the Deep Valley site was lower compared to among‐site variability (13–49% lower in Medium Valley and 47–80% lower in Deep Valley, depending on season) (Table S3).

### 3.2 Hourly cold‐air pooling dynamics

**FIGURE 3:**
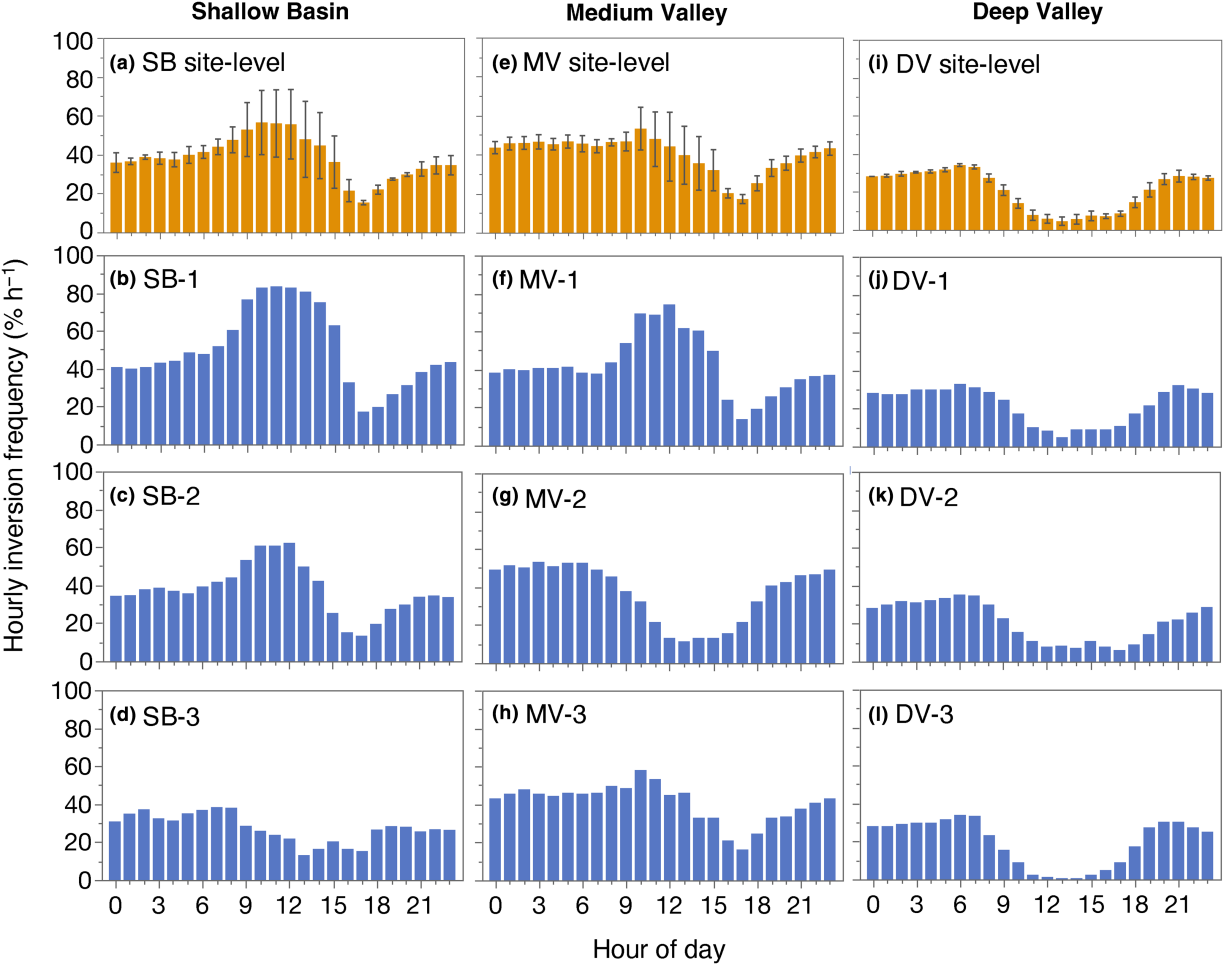
Hourly inversion frequency at the site‐level and transect‐level (in percent of dates over the full shared fall–winter study period for each hourly interval). Transect‐level frequencies are shown in the bottom three rows (blue), while the top row (orange) shows site‐level means ±1 SE of the 3 transect frequencies per hour. (a–d) Shallow Basin; (e–h) Medium Valley; (i–l) Deep Valley. The shared study period is September 25, 2021—March 12, 2022.

We found that site‐level hourly inversion frequencies (i.e., percentage of dates with inversion presence for a given hourly interval) ranged from 5 to 56%, showing that inversions can occur any hour (Figure 3a,e,i). Additionally, our data show that predominant diurnal patterns of inversion frequency (i.e., the times during the day/night inversions were most frequent) were variable across, and in some cases within, sites (Figure 3, Table S6). In Deep Valley, inversions at all transects were most frequent during the evening and night (between 3 pm and 7 am EST; Figure 3i–l, Table S6). In contrast, within‐site variability in the timing of inversions was high in the Shallow Basin and Medium Valley sites, with some transects having strong and frequent inversions during the day (9 am–4 pm EST; Figure 3a–h, Table S6). Indeed, inversions were more frequent during the day than night at SB‐1, SB‐2, and MV‐1, reaching daytime hourly frequencies of 44–71%, some of the highest hourly inversion frequencies we observed across all three sites (Figure 3).

**FIGURE 4:**
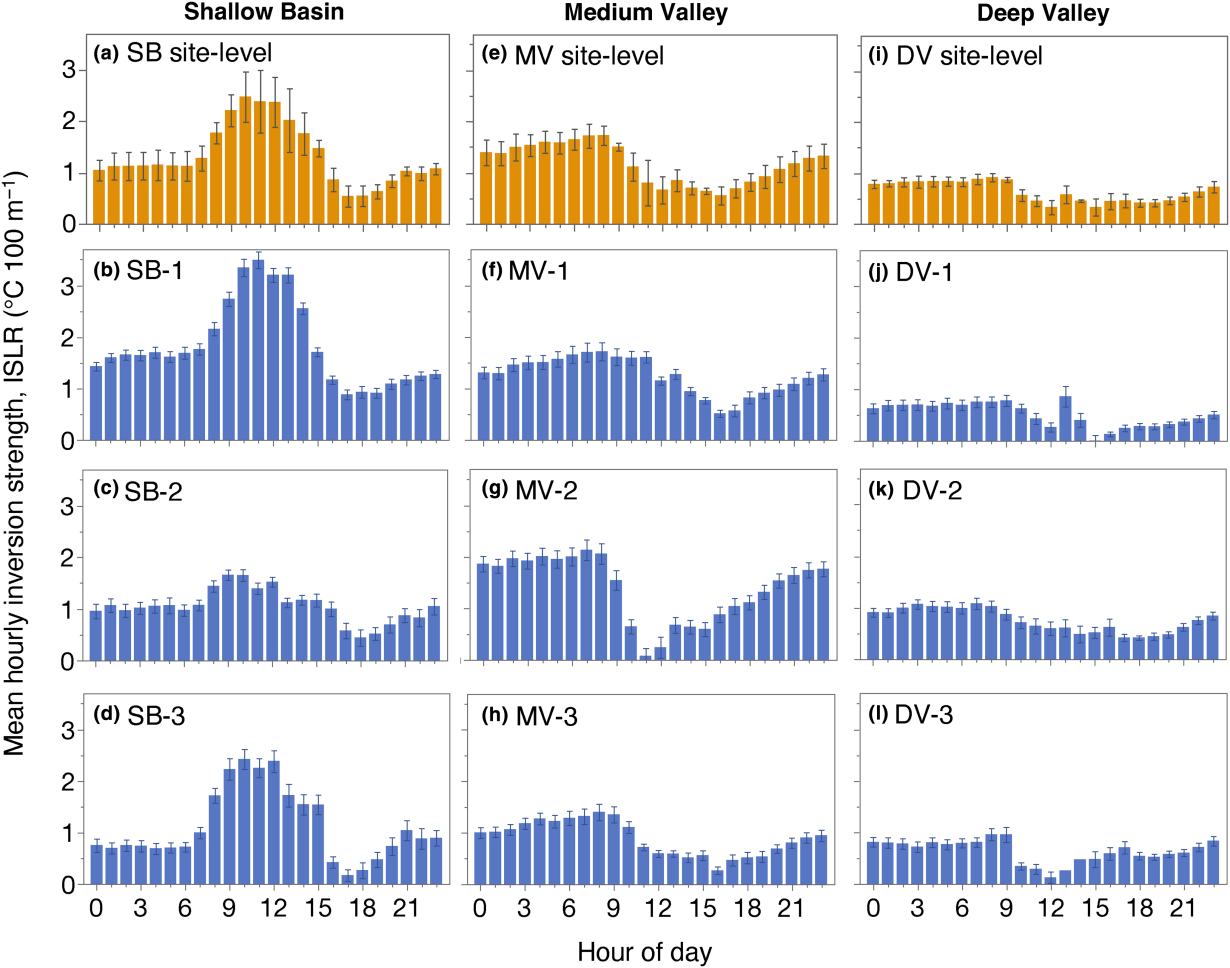
Mean hourly inversion strength (ISLR) at the site‐level and transect‐level. Transect‐level mean inversion strength (bottom three rows in blue) was calculated as the mean decline in air temperature with decreasing elevation across dates over the full shared fall–winter study period for each hourly interval, on a standardized scale (units of °C 100 m^−1^). The top row (orange) shows site‐level means ±1 SE of the 3 transect means. (a–d) Shallow Basin; (e–h) Medium Valley; (i–l) Deep Valley. Error bars = ± 1 SE. The shared study period is September 25, 2021–March 12, 2022.

Our data show steep elevational temperature gradients during inversions at all hours (Figure 4, Table S7). Overall, at the site‐level we found that mean hourly inversion strength ranged from 0.3 to 2.5°C 100 m^−1^ (Figure 4a,e,i) and was positively correlated with hourly inversion frequency (Figure S2), although there was considerable scatter, indicating that hourly inversion frequency could not always predict hourly inversion strength. Yet, like hourly inversion frequency patterns, we observed higher among‐site than within‐site variability in hourly inversion strength for the Deep Valley site but less consistency for the Shallow Basin and Medium Valley sites (Table S6).

Inversions were stronger, on average, at night than during the day at the Deep and Medium Valley sites (Figure 4e–i). In contrast, at the Shallow Basin site, inversions were stronger during the day than night, but with considerable variation in hourly inversion strength among transects (Figure 4a–d, Table S6). Moreover, we found that daytime inversions at SB‐1 were markedly stronger than inversions at any other transect (Figure 4b). Notably, daytime inversions at SB‐3 were among the strongest observed at any transect (Figure 4d) despite being modestly less frequent than nighttime inversions (Figure 3d).

### 3.3 Edaphic properties

We did not observe significant trends in soil nitrogen concentration, soil pH, or gravimetric soil water content across elevation at any site (Figure S3). These three properties were similar at Deep Valley and Shallow Basin, but at Medium Valley, on average across plots, soil nitrogen concentration was 19% higher, soil pH was 9% lower, and gravimetric soil water content was 41% higher than at the other sites. Soil depth did not change with elevation at Shallow Basin or Medium Valley, but decreased with increasing elevation at Deep Valley (*p* = .046, *R* ^2^ = 0.2, Figure S3). On average, soil depth was 35 cm at Medium Valley, 49 cm at Deep Valley, and 56 cm at Shallow Basin.

### 3.4 Forest composition patterns

**FIGURE 5:**
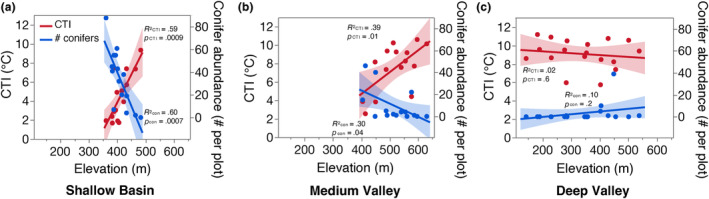
Change in forest overstory community temperature index (CTI; community weighted mean of species' preferred temperatures) and conifer abundance per plot with elevation at (a) Shallow Basin, (b) Medium Valley, and (c) Deep Valley. Each point represents one plot. Shaded areas = 95% CI of linear regressions. Understory communities are shown in Figure S4 and linear regressions for warm‐broadleaved and cold‐broadleaved overstory abundances are in Figure S5.

We found that the community temperature index (CTI) rapidly decreased with declining elevation (i.e., inverted CTI patterns) at the Medium Valley and Shallow Basin sites, which both displayed notably more frequent and stronger cold‐air pooling than the Deep Valley site (Figure 5, Figures S4, S5). The CTI trends were similar in the overstory and understory communities (*R* ^2^ = 0.52 for linear regression of understory vs. overstory CTI); however, we only observed significant decreases in CTI with declining elevation in the overstory communities (Figure 5, Figure S4). The inverted overstory CTI patterns were largely driven by increases in conifer abundance with decreasing elevation, particularly at the Shallow Basin site where low‐elevation conifer abundance was especially high (Figure 5, Figures S3, S5, Table S1). The coniferous group at the Shallow Basin and Medium Valley sites was mostly composed of *A. balsamea* and *Picea* species (Table S1). Decreases in warm‐broadleaved species with decreasing elevation also contributed to the overstory CTI patterns, but to a lesser extent (Figure S5), and were most notable in the understory community at the Shallow Basin site (Figure S4). Our data show that *A. saccharum*, *Acer rubrum*, and *F. grandifolia* dominated the warm‐broadleaved group (Table S1). The abundance of cold‐broadleaved trees (dominated by *Betula* species and, in the understory, also *Acer pensylvanicum*, Table S1) did not significantly change with elevation at any site (Figures S4, S5). CTI and tree functional group abundances did not change with elevation at the Deep Valley site (Figure 5, Figures S4, S5), which experienced less frequent and weaker cold‐air pooling compared to the other two sites.

## 4 DISCUSSION

Cold‐air pooling is likely a fundamental ecological process that drives forest structure and function in regions with complex topography. We characterized temporal cold‐air pooling dynamics and explored links to forest composition at local to regional scales in New England. We hypothesized that cold‐air pooling would affect forest composition by favoring cold‐preference tree species at low elevations, leading to inverted forest composition patterns with elevation. We found that cold‐air pooling was frequent and strong at all sites, but variable at both local and regional scales. Interestingly, we observed cold‐air pooling in all seasons that we measured and all hours of the day and night, indicating that the traditional view of cold‐air pooling as a nocturnal process favored in winter does not apply at fine scales and/or beneath the forest canopy. Importantly, we observed inverted forest composition patterns across elevation where cold‐air pooling was most frequent and strongest, most notably in the overstory communities, supporting our hypothesis that cold‐air pooling may play a key role in structuring forest communities across New England. We discuss these results in detail below.

### 4.1 Frequent and strong cold‐air pooling

Our results revealed frequent cold‐air pooling in all seasons measured across the New England region (19–43% occurrence) with inversion strength ranging from 0.5–1.7°C 100 m^−1^ on average (depending on site and season). The standard environmental lapse rate is −0.65°C 100 m^−1^ (Barry & Chorley, 2010); cold‐air pooling not only changed the direction of the lapse rate (i.e., from negative to positive, as expected) but also led to changes in temperature with elevation that were often more than 2.5× greater in magnitude than the standard environmental lapse rate. Thus, simple elevation‐based lapse rate models based on a well‐mixed atmosphere may produce poor temperature estimates during times of high atmospheric stability that promote cold‐air pooling (Daly, 2006; Dobrowski et al., 2009). Studies across other geographic regions and landforms have also observed frequent cold‐air pooling (≈20–80% of the time) with strong temperature gradients (Blandford et al., 2008; Dobrowski, 2011; Dobrowski et al., 2009; Duine et al., 2017; Jemmett‐Smith et al., 2018; Rupp et al., 2020; Samways, 1990). Despite the ubiquity of frequent and strong cold‐air pooling across sites in our study, we observed a surprising amount of local variation.

Many past topoclimate studies have focused on either small areas (e.g., single watershed) or broad extents/large spatial grains (Fridley, 2009). Here, we captured both scales by characterizing cold‐air pooling at multiple sites across a broad region but at small transect scales and in the near‐ground environment that organisms experience. We found that among‐site (regional) variability in cold‐air pooling frequency and strength was in some cases higher and in other cases lower than within‐site (local) variability. Synoptic‐scale weather conditions (e.g., anticylonic flows that produce high pressure, stable conditions) and proximity to water bodies may have contributed to variability in cold‐air pooling across the broad region (Dobrowski et al., 2009; Fridley, 2009). The presence of Lake Champlain and the broader river valley may have led to less widely fluctuating temperatures at the Deep Valley site and hence weaker and less frequent cold‐air pooling compared to the other two sites. Additionally, the main valley at the Deep Valley site is oriented similarly to the prevailing wind direction (west‐northwest; Global Wind Atlas, 2023), which may allow air to quickly drain down the valley and lead to less frequent cold‐air pooling with weaker temperature inversions. The within‐site variability we observed was likely due to local‐scale factors like wind direction and fine‐scale physiographic features such as curvature or aspect (Dobrowski et al., 2009). Our finding of heterogeneous topoclimatic microenvironments is important because they may support diverse ecosystem structures and functions across local areas. Although there have not been many studies characterizing cold‐air pooling at both local and regional scales, one study in mountain valleys of the Yukon, Canada, found more synchronicity of cold‐air pooling among transects within a local site compared to across sites (Noad & Bonnaventure, 2022). Clearly, we need more studies to understand spatial variation in cold‐air pooling dynamics.

We observed some unexpected seasonal and diurnal cold‐air pooling dynamics that contrast with common views of cold‐air pooling as a nocturnal, wintertime process (Jemmett‐Smith et al., 2018; Noad & Bonnaventure, 2022; Reeves & Stensrud, 2009; Whiteman et al., 1999, 2001, 2010; Wolyn & McKee, 1989). For instance, the frequency of cold‐air pooling was relatively similar among seasons on average and was opposite of expectations for the only site (Deep Valley) that did show relatively consistent seasonal patterns; that is, we found the highest frequencies in late summer and the lowest in winter. Other studies have also observed frequent cold‐air pooling in summer and fall (Bigg et al., 2014; Iijima & Shinoda, 2000; Novick et al., 2016; Pypker, Unsworth, Mix, et al., 2007), indicating that this process is not limited to winter and seasonal dispositions vary among sites. In addition, although inversions were generally more frequent during the night than the day, we found the opposite pattern at three transects that experienced some of the strongest inversions. The differences between our study and others may be related to scale. Our measurements occurred across ≈1–5‐km transects while other studies characterized cold‐air pooling using distant weather stations or with coarse‐scale gridded temperature data from remote sensing. Moreover, our sensors were located below the forest canopy, whereas most temperature measurements are taken above the canopy or in unforested areas (De Frenne & Verheyen, 2016). Inversions beneath the forest canopy may form during the day and persist longer because of contact with the cold ground, friction from understory vegetation, and sheltering from insolation and wind, sometimes leading to wind flowing up‐valley above the canopy and down‐valley below the canopy on summer days (Kelsey et al., 2019; Kiefer & Zhong, 2013; Pypker, Unsworth, Lamb, et al., 2007; Rupp et al., 2020). We predict that daytime cold‐air pools may be most common where the canopy is densest, an idea that should be tested in future studies.

### 4.2 Cold‐air pooling influences forest composition

We established that cold‐air pooling is common and generates strong temperature gradients capable of impacting ecosystem dynamics across the New England region. Indeed, we found compelling evidence linking cold‐air pooling to changes in forest composition across slopes. Specifically, we observed forest composition inversions at the two sites (Shallow Basin and Medium Valley) with the highest frequency and intensity of temperature inversions, supporting our hypothesis. At those two sites, the community temperature index (CTI) increased with elevation, indicating that the preferred temperature of the forest community increased with elevation. In contrast, traditional climatic gradients across slopes (i.e., those similar to standard environmental lapse rates) typically lead to decreasing CTI and greater abundances of cold‐loving species with increasing elevation (Cogbill & White, 1991; Kupfer & Cairns, 1996; Savage & Vellend, 2015; Siccama, 1974). The inverted CTI patterns we observed resulted from more cold‐loving conifers (*A. balsam* and *Picea* spp.) at low compared to high elevations at the two sites where cold‐air pooling was most frequent, as predicted. To a lesser extent, more warm‐loving broadleaved species (*F. grandifolia* and *Acer* spp.) at high compared to low elevations also contributed to the pattern.

We observed stronger CTI inversions in the overstory than the understory forest communities, likely reflecting the closed canopy, second‐growth nature of these forests. The composition of the present overstory was likely shaped by cold‐air pooling in the past when the now‐mature trees were initially establishing as vulnerable seedlings in exposed conditions with little or no cover (Blennow & Lindkvist, 2000), particularly at the Shallow Basin site, which experienced historic, intensive disturbance from management. However, as the trees grew they modified the sub‐canopy microenvironment that dictates species regeneration, such as by reducing light availability and generating a thick organic soil layer. Most of the conifer species dominating the canopy of the sites with frequent cold‐air pooling rely on well‐decomposed wood or exposed mineral soil and some light availability (>5%) for establishment and recruitment (Weaver et al., 2009). Therefore, current conditions may not be ideal for regeneration of these cold‐dependent species, but may become suitable as forest development proceeds and single‐tree and multiple‐tree fall gaps are created in the canopy by the common mortality agents for these systems (wind, insects, and disease; Worrall et al., 2005). Thus, as suitable conifer regeneration sites become available in the future, cold‐air pooling may again alleviate competitive pressure for conifer establishment. An alternative explanation for the stronger cold‐air pooling signature in the overstory community is that the understory is buffered from cold‐air pooling by the canopy; however, our measurements of cold‐air pooling occurred beneath the overstory canopy at 1.5 m above the ground surface. It is possible that smaller saplings and seedlings experienced some buffering from sub‐canopy vegetation beneath our sensors, depending on forest structure, and from insulating snowpack during the snow season (Drescher & Thomas, 2013). To better understand relationships between cold‐air pooling and regeneration, we need cold‐air pooling studies that are long‐term or coordinated studies across forest developmental stages.

In addition to disrupting climatic gradients, cold‐air pooling could interact with biophysical gradients to influence forest composition. Decreases in soil depth with increasing elevation typically favor conifer dominance along upper slopes in the New England region (Leak, 1991; Lee et al., 2005). However, we only observed decreasing soil depth with increasing elevation at Deep Valley, where CTI did not shift across elevation, and there were no changes in soil pH, soil nitrogen, or point measurements of soil moisture across elevation at any site. Although we did not find evidence that cold‐air pooling microenvironments store more soil resources, valley floors where cold‐air pooling occurs can retain soil moisture (Frei et al., 2023) and cold‐air pooling itself can influence dew formation and vapor pressure deficit and could indirectly influence soil moisture via effects on plant ecophysiological processes (Hubbart et al., 2007; McLaughlin et al., 2017; Novick et al., 2016). In the future, high‐frequency soil moisture measurements across seasons would allow us to better assess potential interactions between cold‐air pooling, topography, and soil moisture to determine whether these areas could act as microrefugia not only by providing relatively cooler conditions but also greater water availability.

## 5 CONCLUSIONS

Overall, we found that cold‐air pooling is a common but spatially variable phenomenon capable of generating strong temperature inversions and influencing forest composition. Our finding of frequent and strong inversions during the day and across seasons is different than studies that characterized inversions at coarser scales, above forest canopies, or in unforested areas. Such discrepancies suggest that fine‐scale topography and/or forest canopies shelter inversions from the conditions (e.g., solar heating, wind movement) that break inversions up, allowing for frequent and strong inversions beneath forest canopies at transect scales (i.e., 1–5 km). Topoclimatic, physiographic, and biophysical features may interact to create multiple decoupled air layers, and thus we need studies that simultaneously compare cold‐air pooling dynamics near the ground surface, within the dense foliage zone, and above the canopy.

The presence of daytime cold‐air pooling and thus reduced maximum daytime air temperatures in the biotic zone may also allow these areas to mitigate some of the effects of climate change (Rupp et al., 2020). Although areas prone to cold‐air pooling are unlikely to escape regional climate change, cold‐air pools are decoupled from the free atmosphere and thus may respond differently to macroclimate warming than the broader region (Daly et al., 2010; Vitasse et al., 2017), allowing them to serve as microrefugia for cold‐loving species and associated ecosystem functions. Indeed, our finding that cold‐air pooling influences forest composition suggests that cold‐air pooling could affect key ecosystem functions, such as carbon storage, water filtration, snow retention, and habitat provisioning, via climate‐plant–soil feedbacks. For instance, cold‐air pooling may promote soil carbon accrual and storage in low‐elevation pockets by favoring and protecting cold‐loving conifers that are vulnerable to climate change. Conifers are associated with slow soil organic carbon turnover (Boča et al., 2014; Cornwell et al., 2008; Jevon et al., 2019) and could reinforce the cool, wet microenvironments found in cold‐air pooling areas (Pastore et al., 2022). Thus, a better understanding of cold‐air pooling dynamics and associated ecological impacts will improve conservation planning focused on sustaining cold‐dependent species and functions. This includes better identifying potential climate refugia for species projected to regionally decline under a warmer climate (Morelli et al., 2016).

In summary, cold‐air pooling may act as a fundamental ecological process that drives ecosystem structure and function, as well as modulates ecosystem responses to warming. Moving forwards, we need cold‐air pooling studies that capture interannual weather variability and studies in more locations to determine whether the cold‐air pooling dynamics we observed within the sub‐canopy are a regional phenomenon versus more widespread. Studies in more locations will also help us draw stronger conclusions about the role cold‐air pooling plays in structuring forest composition. Additional spatiotemporal characterizations of cold‐air pooling within the biotic zone are critically needed for understanding and predicting the impacts of this often overlooked phenomenon on plant communities, wildlife, soil processes, and other important ecosystem dynamics.

## AUTHOR CONTRIBUTIONS

**Melissa A. Pastore:** Conceptualization (lead); data curation (equal); formal analysis (lead); investigation (equal); methodology (lead); project administration (equal); supervision (lead); validation (equal); visualization (lead); writing – original draft (lead); writing – review and editing (lead). **Aimée T. Classen:** Conceptualization (equal); funding acquisition (equal); methodology (supporting); project administration (supporting); resources (equal); supervision (supporting); validation (equal); writing – review and editing (equal). **Anthony W. D'Amato:** Conceptualization (equal); funding acquisition (equal); methodology (supporting); project administration (supporting); resources (equal); supervision (supporting); validation (equal); writing – review and editing (equal). **Marie E. English:** Data curation (equal); investigation (equal); methodology (supporting); project administration (supporting); validation (equal); writing – review and editing (equal). **Karin Rand:** Data curation (equal); investigation (equal); methodology (supporting); project administration (supporting); validation (equal); writing – review and editing (equal). **Jane R. Foster:** Conceptualization (equal); data curation (equal); methodology (supporting); project administration (supporting); validation (equal); writing – review and editing (equal). **E. Carol Adair:** Conceptualization (equal); funding acquisition (equal); methodology (supporting); project administration (supporting); resources (equal); supervision (lead); validation (equal); writing – review and editing (equal).

## CONFLICT OF INTEREST STATEMENT

The authors declare no conflict of interest.

## Supporting information


Appendix S1.

## Data Availability

Data are available from the Environmental Data Initiative (EDI): https://doi.org/10.6073/pasta/d78f0f01a24c3590d591cc8c32a9c6c3 (Pastore et al., 2024).
