# Supplementary material for: Frequent and strong cold‐air pooling drives temperate forest composition
Source: Ecol Evol. 2024 Apr 1;14(4):e11126. doi: 10.1002/ece3.11126 (PMC10985370; doi:10.1002/ece3.11126)
Supplement: Supplementary file 1 — Appendix S1. [file ECE3-14-e11126-s001.docx]

**Supplementary Information**

**Ecology and Evolution**

**Frequent and strong cold-air pooling drives temperate forest composition**

Melissa A. Pastore^*^, Aimée T. Classen, Anthony W. D’Amato, Marie E. English, Karin Rand, Jane R. Foster, E. Carol Adair

*Correspondence to: [Melissa.Pastore@usda.gov](mailto:Melissa.Pastore@usda.gov)

**Figure S1.** Seasonal cold-air pooling dynamics at the site-level (a-b) and transect-level (c-d: Shallow Basin; e-f: Medium Valley; g-h: Deep Valley). Top row: Mean transect temperature difference during inversions by season. Bottom row: Maximum transect temperature difference during inversions by season. Temperature difference was calculated by subtracting the temperature of the first/lowest plot from that of the last/highest plot in a transect and was not standardized by elevation (i.e., this figure shows temperature differences across entire transects that vary in elevation). Each seasonal site-level bar in a-b is the mean ± 1 SE of 3 transects. Each seasonal transect-level bar in c, e, g is the mean ± 1 SE across all hourly timesteps when an inversion was present within a given season. Note that data from Nulhegan Basin are only available from fall and winter.


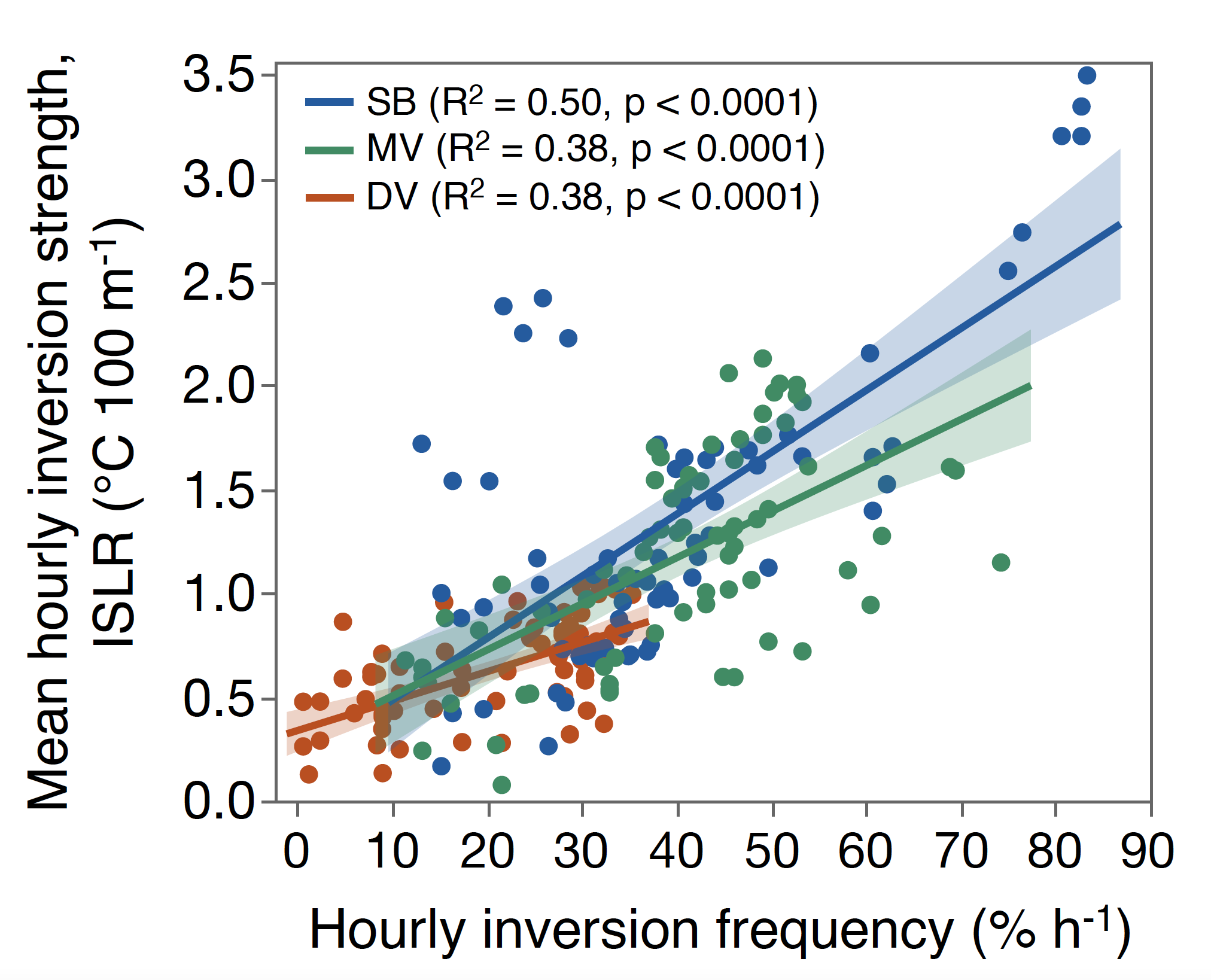


**Figure S2.** Relationships between hourly inversion frequency and mean hourly inversion strength (ISLR) for each site (SB = Shallow Basin; MV = Medium Valley; DV = Deep Valley). Each point represents one hourly interval for one transect. Shaded areas = 95% CI of linear regressions.


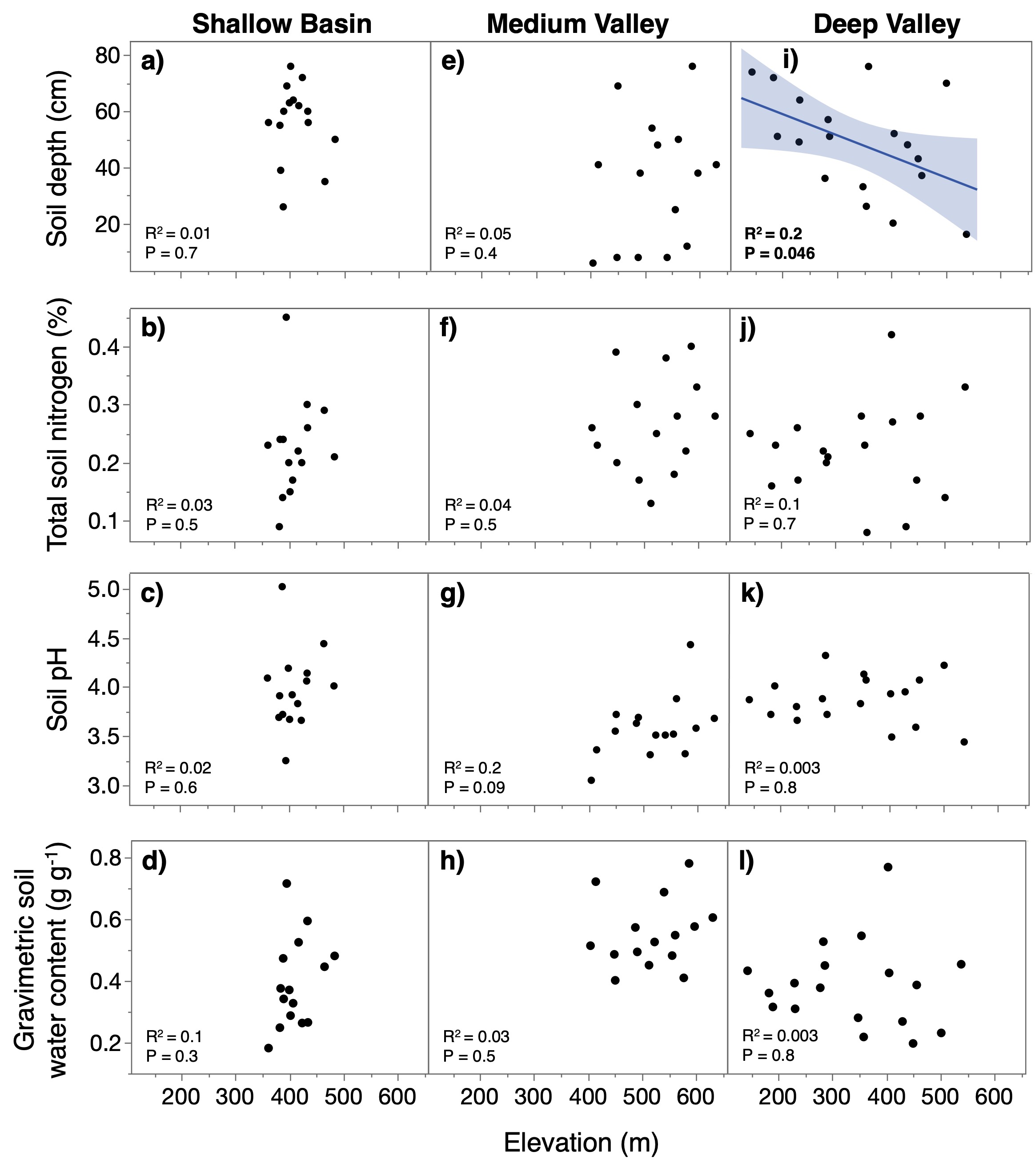
**Figure S3.** Soil edaphic properties for each plot across elevation, grouped by site: (a-d) Shallow Basin, (e-h) Medium Valley, and (i-l) Deep Valley. Each point represents one plot. Linear regression with 95% CI (shaded area) shown when p<0.05. We only observed one significant relationship, which was a decline in soil depth with increasing elevation at Deep Valley (i).


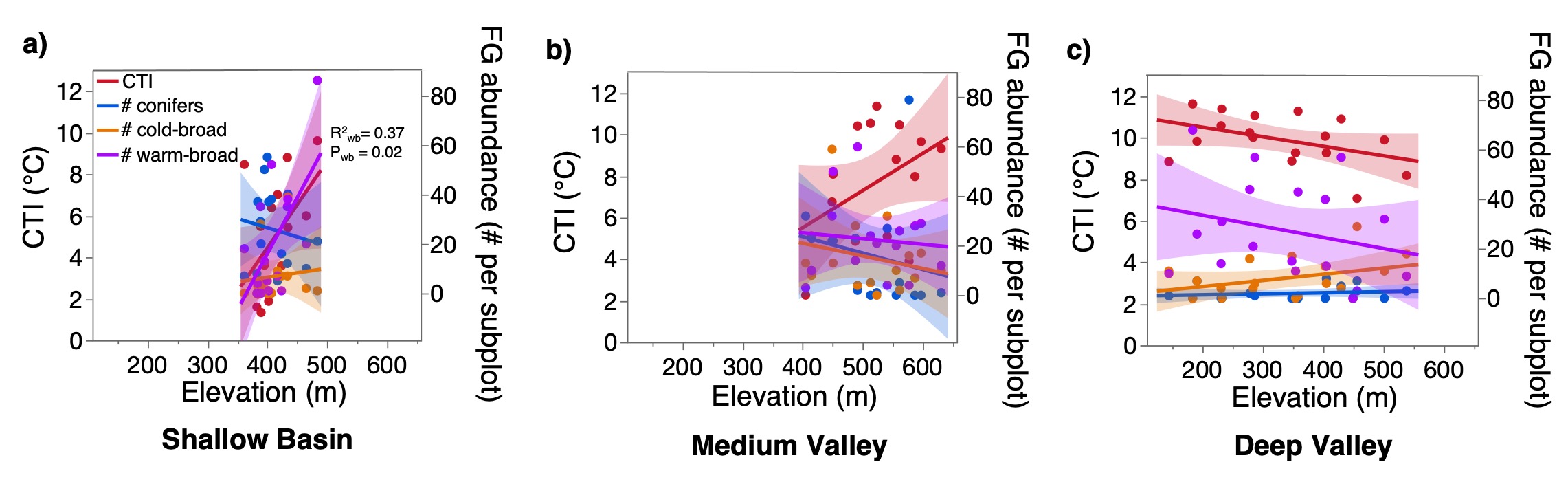


**Figure S4.** Change in forest understory community temperature index (CTI; community weighted mean of species’ preferred temperatures) and abundances of coniferous, cold-broadleaved, and warm-broadleaved tree functional groups (FG) with elevation at (a) Shallow Basin, (b) Medium Valley, and (c) Deep Valley. Each point represents one plot. Shaded areas = 95% CI of linear regressions. R^2^ and p-values are shown only for linear regressions in which p<0.05 (i.e., warm-broadleaved ‘wb’ species in panel a).


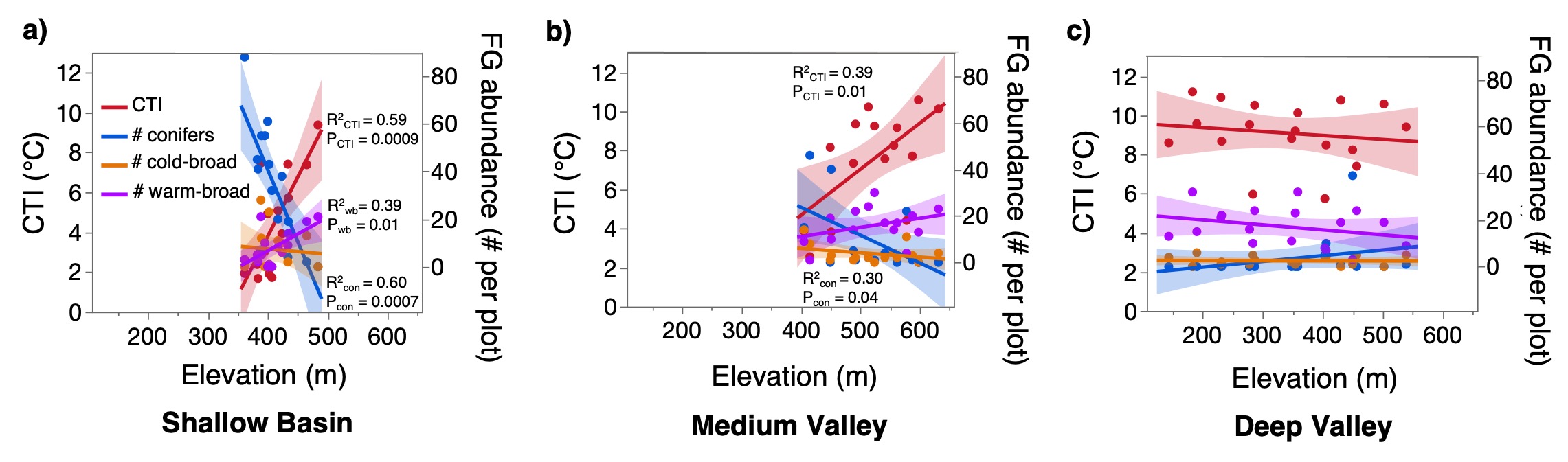


**Figure S5.** Change in forest overstory community temperature index (CTI; community weighted mean of species’ preferred temperatures) and abundances of coniferous, cold-broadleaved, and warm-broadleaved tree functional groups (FG) with elevation at (a) Shallow Basin, (b) Medium Valley, and (c) Deep Valley. Each point represents one plot. Shaded areas = 95% CI of linear regressions. R^2^ and p-values are shown only for linear regressions in which p<0.05 (‘CTI’ subscript corresponds to CTI in red; ‘wb’ subscript corresponds to warm-broadleaved in purple; ‘con’ subscript corresponds to conifers in blue).

**Table S1.** Preferred temperatures and functional groups of the tree species observed. Species abundances are listed for each site. Overstory plot (top) and understory sub-plot (bottom) abundances are shown separately.

| **OVERSTORY** | | | | | |
| --- | --- | --- | --- | --- | --- |
| Tree functional group | Species | Preferred temperature °C | Species abundance (# of trees) | | |
|  |  |  | Shallow Basin | Medium Valley | Deep Valley |
| Coniferous | Tamarack (*Larix laricina*) | -0.19 | 6 | 0 | 0 |
| Coniferous | Balsam Fir (*Abies balsamea*) | 1.48 | 311 | 81 | 5 |
| Coniferous | Black/Red Spruce (*Picea mariana/Picea rubens*) | 2.01 | 138 | 0 | 0 |
| Coniferous | Red Spruce (*Picea rubens*) | 5.85 | 45 | 60 | 3 |
| Coniferous | Eastern White Pine (*Pinus strobus*) | 6.55 | 6 | 0 | 0 |
| Coniferous | Eastern Hemlock (*Tsuga canadensis*) | 8.06 | 4 | 0 | 49 |
| Cold-broadleaved | Mountain Paper Birch (*Betula cordifolia*) | -0.52 | 0 | 5 | 0 |
| Cold-broadleaved | Paper Birch (*Betula papyrifera*) | -0.52 | 20 | 28 | 16 |
| Cold-broadleaved | Quaking Aspen (*Populus tremuloides*) | 0.50 | 10 | 2 | 0 |
| Cold-broadleaved | Yellow Birch (*Betula alleghaniensis*) | 6.41 | 67 | 12 | 20 |
| Cold-broadleaved | Bigtooth Aspen (*Populus grandidentata*) | 6.55 | 0 | 0 | 3 |
| Cold-broadleaved | Striped Maple (*Acer pensylvanicum*) | 6.91 | 4 | 10 | 7 |
| Cold-broadleaved | Gray Birch (*Betula populifolia*) | 7.07 | 8 | 0 | 0 |
| Warm-broadleaved | Sugar Maple (*Acer saccharum*) | 8.23 | 41 | 131 | 102 |
| Warm-broadleaved | American Basswood (*Tilia americana*) | 8.69 | 0 | 0 | 4 |
| Warm-broadleaved | Serviceberry (*Amelanchier spp.*) | 9.44 | 0 | 0 | 1 |
| Warm-broadleaved | Northern Red Oak (*Quercus rubra*) | 9.84 | 0 | 0 | 23 |
| Warm-broadleaved | Black Birch (*Betula lenta*) | 10.35 | 2 | 0 | 0 |
| Warm-broadleaved | American Hophornbeam (*Ostrya virginiana*) | 10.95 | 0 | 0 | 7 |
| Warm-broadleaved | Red Maple (*Acer rubrum*) | 11.19 | 63 | 34 | 32 |
| Warm-broadleaved | American Beech (*Fagus grandifolia*) | 11.64 | 11 | 71 | 124 |
| Warm-broadleaved | White Ash (*Fraxinus americana*) | 11.78 | 2 | 0 | 11 |
| Warm-broadleaved | Black Cherry (*Prunus serotina*) | 13.01 | 5 | 1 | 4 |
| **UNDERSTORY** | | | | | |
| Tree functional group | Species | Preferred temperature °C | Species abundance (# of trees) | | |
|  |  |  | Shallow Basin | Medium Valley | Deep Valley |
| Coniferous | White Spruce (*Picea glauca*) | -1.55 | 0 | 1 | 0 |
| Coniferous | Tamarack (*Larix laricina*) | -0.19 | 10 | 0 | 0 |
| Coniferous | Balsam Fir (*Abies balsamea*) | 1.48 | 277 | 178 | 0 |
| Coniferous | Black/Red Spruce (*Picea mariana/Picea rubens*) | 2.01 | 96 | 0 | 0 |
| Coniferous | Northern White Cedar (*Thuja occidentalis*) | 3.85 | 2 | 0 | 0 |
| Coniferous | Red Spruce (*Picea rubens*) | 5.85 | 0 | 58 | 18 |
| Coniferous | Eastern Hemlock (*Tsuga canadensis*) | 8.06 | 0 | 0 | 18 |
| Cold-broadleaved | Mountain Paper Birch (*Betula cordifolia*) | -0.52 | 0 | 6 | 0 |
| Cold-broadleaved | Paper Birch (*Betula papyrifera*) | -0.52 | 7 | 6 | 0 |
| Cold-broadleaved | Balsam Poplar (*Populus balsamifera*) | -0.48 | 30 | 0 | 0 |
| Cold-broadleaved | American Mountain Ash (*Sorbus americana*) | 2.86 | 0 | 2 | 0 |
| Cold-broadleaved | Mountain Maple (*Acer spicatum*) | 4.39 | 0 | 24 | 0 |
| Cold-broadleaved | Black Ash (*Fraxinus nigra*) | 5.65 | 1 | 0 | 0 |
| Cold-broadleaved | Yellow Birch (*Betula alleghaniensis*) | 6.41 | 34 | 40 | 15 |
| Cold-broadleaved | Bigtooth Aspen (*Populus grandidentata*) | 6.55 | 0 | 0 | 8 |
| Cold-broadleaved | Striped Maple (*Acer pensylvanicum*) | 6.91 | 29 | 143 | 124 |
| Cold-broadleaved | Gray Birch (*Betula populifolia*) | 7.07 | 2 | 3 | 0 |
| Warm-broadleaved | Sugar Maple (*Acer saccharum*) | 8.23 | 44 | 85 | 30 |
| Warm-broadleaved | Serviceberry (*Amelanchier spp.*) | 9.44 | 42 | 0 | 5 |
| Warm-broadleaved | Northern Red Oak (*Quercus rubra*) | 9.84 | 0 | 0 | 4 |
| Warm-broadleaved | American Hophornbeam (*Ostrya virginiana*) | 10.95 | 0 | 0 | 4 |
| Warm-broadleaved | Red Maple (*Acer rubrum*) | 11.19 | 75 | 71 | 16 |
| Warm-broadleaved | American Beech (*Fagus grandifolia*) | 11.64 | 120 | 166 | 433 |
| Warm-broadleaved | White Ash (*Fraxinus americana*) | 11.78 | 8 | 1 | 2 |
| Warm-broadleaved | Black Cherry (*Prunus serotina*) | 13.01 | 26 | 14 | 0 |

**Table S2.** Monthly inversion frequency (in % of time per month) at the site-level (mean ± 1 SE of 3 transects) and transect-level. Monthly inversion frequency was calculated as the percentage of hourly timesteps per month that an inversion was present. Only months with complete data are shown (see Table 1 for measurement dates).

| Shallow Basin | | | | | |
| --- | --- | --- | --- | --- | --- |
|  | Site-level | | SB-1 | SB-2 | SB-3 |
| Month | Mean frequency (%) | SE | Frequency (%) | Frequency (%) | Frequency (%) |
| 1 | 34.66 | 7.78 | 48.86 | 33.06 | 22.04 |
| 2 | 37.97 | 7.90 | 52.68 | 35.64 | 25.60 |
| 10 | 46.89 | 7.85 | 52.15 | 57.06 | 31.45 |
| 11 | 42.38 | 5.50 | 53.33 | 36.04 | 37.78 |
| 12 | 28.52 | 7.62 | 43.75 | 21.37 | 20.43 |
| Medium Valley | | | | | |
|  | Site-level | | MV-1 | MV-2 | MV-3 |
| Month | Mean frequency (%) | SE | Frequency (%) | Frequency (%) | Frequency (%) |
| 1 | 44.67 | 2.04 | 46.77 | 46.64 | 40.59 |
| 2 | 42.86 | 4.50 | 48.96 | 45.54 | 34.08 |
| 3 | 37.77 | 6.19 | 44.62 | 43.28 | 25.40 |
| 4 | 39.77 | 7.54 | 51.94 | 41.39 | 25.97 |
| 5 | 50.67 | 6.99 | 61.29 | 53.23 | 37.50 |
| 6 | 40.19 | 2.62 | 43.47 | 42.08 | 35.00 |
| 9 | 44.07 | 5.89 | 37.50 | 38.89 | 55.83 |
| 10 | 36.02 | 4.68 | 38.31 | 27.02 | 42.74 |
| 11 | 43.68 | 1.38 | 44.43 | 45.62 | 41.01 |
| 12 | 34.41 | 2.90 | 35.62 | 38.71 | 28.90 |
| Deep Valley | | | | | |
|  | Site-level |  | DV-1 | DV-2 | DV-3 |
| Month | Mean frequency (%) | SE | Frequency (%) | Frequency (%) | Frequency (%) |
| 1 | 25.00 | 1.09 | 25.00 | 26.88 | 23.12 |
| 2 | 12.85 | 1.00 | 14.73 | 12.50 | 11.31 |
| 3 | 15.55 | 1.67 | 18.55 | 15.32 | 12.77 |
| 4 | 17.73 | 2.34 | 21.53 | 13.47 | 18.19 |
| 5 | 31.18 | 0.87 | 29.44 | 31.99 | 32.12 |
| 8 | 28.56 | 2.08 | NA | 26.48 | 30.65 |
| 9 | 27.01 | 2.75 | 32.50 | 24.58 | 23.96 |
| 10 | 23.46 | 2.93 | 29.12 | 21.95 | 19.31 |
| 11 | 27.55 | 2.13 | 25.42 | 25.42 | 31.81 |
| 12 | 13.75 | 2.13 | 11.56 | 18.01 | 11.69 |

**Table S3.** Among- vs. within-site variability in seasonal inversion frequency and strength (ISLR). Within-site variability of each site is 1 SE of the mean across all 3 transects per site. Among-site variability is 1 SE of the mean across all 3 sites.

|  |  | Among-site variability | Within-site variability | | |
| --- | --- | --- | --- | --- | --- |
| Variable | Season |  | Shallow Basin | Medium Valley | Deep Valley |
| Seasonal inversion frequency | Fall 2021 | 5.46 | 5.98 | 1.04 | 0.85 |
|  | Late Summer 2021 | 7.11 | NA^a^ | 5.84 | 2.20 |
|  | Spring 2022 | 10.75 | NA^a^ | 6.67 | 0.62 |
|  | Winter 2021/22 | 7.06 | 7.45 | 3.89 | 1.26 |
| Seasonal inversion strength (ISLR) | Fall 2021 | 0.16 | 0.32 | 0.14 | 0.08 |
|  | Late Summer 2021 | 0.26 | NA^a^ | 0.13 | 0.09 |
|  | Spring 2022 | 0.30 | NA^a^ | 0.22 | 0.06 |
|  | Winter 2021/22 | 0.30 | 0.38 | 0.26 | 0.09 |

^a^Data from Shallow Basin are only available from fall and winter.

**Table S4.** Mean inversion strength by month at the site-level and transect-level. Mean inversion strength was calculated as the mean decline in air temperature with decreasing elevation on a standardized scale across transects (ISLR, units of °C 100 m^-1^). Only months with complete data are shown (see Table 1 for measurement dates). We show each site-level mean ± 1 SE of 3 transects and each transect-level mean ± 1 SE across all hourly intervals for each month.

| Shallow Basin | | | | | | | | | |
| --- | --- | --- | --- | --- | --- | --- | --- | --- | --- |
|  | Site-level | | | SB-1 | | SB-2 | | SB-3 | |
| Month | Mean strength  (°C 100 m^-1^) | | SE | Mean strength  (°C 100 m^-1^) | SE | Mean strength  (°C 100 m^-1^) | SE | Mean strength  (°C 100 m^-1^) | SE |
| 1 | 1.43 | | 0.48 | 2.39 | 0.09 | 0.91 | 0.07 | 0.98 | 0.09 |
| 2 | 1.90 | | 0.28 | 2.41 | 0.09 | 1.44 | 0.06 | 1.86 | 0.10 |
| 10 | 1.26 | | 0.32 | 1.90 | 0.05 | 0.95 | 0.05 | 0.92 | 0.06 |
| 11 | 1.32 | | 0.40 | 2.03 | 0.06 | 1.29 | 0.05 | 0.65 | 0.05 |
| 12 | 1.21 | | 0.31 | 1.82 | 0.06 | 0.96 | 0.09 | 0.84 | 0.08 |
| Medium Valley | | | | | | | | | |
|  | | Site-level | | MV-1 | | MV-2 | | MV-3 | |
| Month | | Mean strength  (°C 100 m^-1^) | SE | Mean strength  (°C 100 m^-1^) | SE | Mean strength  (°C 100 m^-1^) | SE | Mean strength  (°C 100 m^-1^) | SE |
| 1 | | 1.62 | 0.31 | 1.59 | 0.07 | 1.09 | 0.06 | 2.18 | 0.10 |
| 2 | | 1.39 | 0.21 | 1.48 | 0.07 | 0.99 | 0.06 | 1.70 | 0.11 |
| 3 | | 1.33 | 0.20 | 1.48 | 0.06 | 0.92 | 0.05 | 1.58 | 0.11 |
| 4 | | 1.22 | 0.20 | 1.41 | 0.06 | 0.82 | 0.05 | 1.42 | 0.08 |
| 5 | | 1.62 | 0.27 | 1.78 | 0.06 | 1.09 | 0.05 | 1.99 | 0.09 |
| 6 | | 0.97 | 0.10 | 0.94 | 0.05 | 0.82 | 0.04 | 1.16 | 0.06 |
| 9 | | 0.99 | 0.14 | 0.99 | 0.04 | 0.76 | 0.04 | 1.23 | 0.05 |
| 10 | | 1.08 | 0.13 | 0.99 | 0.05 | 0.92 | 0.06 | 1.34 | 0.06 |
| 11 | | 1.22 | 0.19 | 1.32 | 0.06 | 0.85 | 0.06 | 1.49 | 0.07 |
| 12 | | 1.21 | 0.16 | 1.16 | 0.06 | 0.96 | 0.06 | 1.52 | 0.10 |
| Deep Valley | | | | | | | | | |
|  | | Site-level | | DV-1 | | DV-2 | | DV-3 | |
| Month | | Mean strength  (°C 100 m^-1^) | SE | Mean strength  (°C 100 m^-1^) | SE | Mean strength  (°C 100 m^-1^) | SE | Mean strength  (°C 100 m^-1^) | SE |
| 1 | | 0.78 | 0.11 | 0.59 | 0.05 | 0.96 | 0.05 | 0.81 | 0.05 |
| 2 | | 0.76 | 0.10 | 0.56 | 0.06 | 0.92 | 0.08 | 0.79 | 0.06 |
| 3 | | 0.73 | 0.09 | 0.54 | 0.05 | 0.84 | 0.06 | 0.81 | 0.08 |
| 4 | | 0.66 | 0.06 | 0.56 | 0.04 | 0.66 | 0.05 | 0.76 | 0.05 |
| 5 | | 0.86 | 0.07 | 0.82 | 0.04 | 0.77 | 0.04 | 0.99 | 0.04 |
| 8 | | 0.71 | 0.03 | NA | NA | 0.69 | 0.03 | 0.74 | 0.02 |
| 9 | | 0.48 | 0.09 | 0.30 | 0.02 | 0.58 | 0.03 | 0.55 | 0.02 |
| 10 | | 0.63 | 0.07 | 0.49 | 0.03 | 0.71 | 0.04 | 0.70 | 0.03 |
| 11 | | 0.98 | 0.08 | 0.90 | 0.06 | 1.15 | 0.06 | 0.91 | 0.05 |
| 12 | | 0.38 | 0.10 | 0.21 | 0.03 | 0.57 | 0.03 | 0.37 | 0.04 |

**Table S5.** Mean transect temperature difference during inversions (T_diff_) by month at the site-level and transect-level. Temperature difference was calculated by subtracting the temperature of the first/lowest plot from that of the last/highest plot in a transect and was not standardized by elevation (i.e., this table shows temperature differences across entire transects that vary in elevation). Only months with complete data are shown (see Table 1 for measurement dates). We show each site-level mean ± 1 SE of 3 transects and each transect-level mean ± 1 SE across all hourly intervals for each month.

| Shallow Basin | | | | | | | | |
| --- | --- | --- | --- | --- | --- | --- | --- | --- |
|  | Site-level | | SB-1 | | SB-2 | | SB-3 | |
| Month | Mean T_diff_ (°C) | SE | Mean T_diff_ (°C) | SE | Mean T_diff_ (°C) | SE | Mean T_diff_ (°C) | SE |
| 1 | 1.48 | 0.61 | 2.67 | 0.10 | 1.07 | 0.04 | 0.69 | 0.04 |
| 2 | 1.53 | 0.54 | 2.61 | 0.10 | 1.13 | 0.05 | 0.86 | 0.05 |
| 10 | 1.14 | 0.42 | 1.98 | 0.06 | 0.76 | 0.03 | 0.68 | 0.03 |
| 11 | 1.20 | 0.49 | 2.17 | 0.07 | 0.86 | 0.03 | 0.57 | 0.03 |
| 12 | 1.17 | 0.43 | 1.99 | 0.07 | 0.98 | 0.05 | 0.52 | 0.04 |
| Medium Valley | | | | | | | | |
|  | Site-level | | MV-1 | | MV-2 | | MV-3 | |
| Month | Mean T_diff_ (°C) | SE | Mean T_diff_ (°C) | SE | Mean T_diff_ (°C) | SE | Mean T_diff_ (°C) | SE |
| 1 | 2.89 | 0.41 | 2.71 | 0.13 | 2.28 | 0.11 | 3.68 | 0.16 |
| 2 | 2.57 | 0.32 | 2.58 | 0.12 | 2.00 | 0.10 | 3.12 | 0.16 |
| 3 | 2.57 | 0.30 | 2.71 | 0.12 | 2.00 | 0.09 | 3.00 | 0.16 |
| 4 | 2.35 | 0.23 | 2.55 | 0.11 | 1.89 | 0.08 | 2.59 | 0.13 |
| 5 | 3.05 | 0.34 | 3.19 | 0.11 | 2.40 | 0.10 | 3.55 | 0.14 |
| 6 | 1.81 | 0.18 | 1.60 | 0.08 | 1.66 | 0.08 | 2.17 | 0.09 |
| 9 | 1.76 | 0.18 | 1.65 | 0.07 | 1.53 | 0.07 | 2.11 | 0.08 |
| 10 | 1.96 | 0.20 | 1.67 | 0.09 | 1.88 | 0.09 | 2.33 | 0.10 |
| 11 | 2.24 | 0.28 | 2.17 | 0.11 | 1.78 | 0.11 | 2.76 | 0.11 |
| 12 | 2.19 | 0.28 | 1.90 | 0.11 | 1.93 | 0.11 | 2.74 | 0.15 |
| Deep Valley | | | | | | | | |
|  | Site-level | | DV-1 | | DV-2 | | DV-3 | |
| Month | Mean T_diff_ (°C) | SE | Mean T_diff_ (°C) | SE | Mean T_diff_ (°C) | SE | Mean T_diff_ (°C) | SE |
| 1 | 2.22 | 0.11 | 2.00 | 0.15 | 2.34 | 0.15 | 2.33 | 0.14 |
| 2 | 2.10 | 0.09 | 2.08 | 0.18 | 2.26 | 0.20 | 1.96 | 0.19 |
| 3 | 2.04 | 0.05 | 1.95 | 0.15 | 2.10 | 0.18 | 2.07 | 0.19 |
| 4 | 1.90 | 0.15 | 1.75 | 0.11 | 1.75 | 0.13 | 2.20 | 0.13 |
| 5 | 2.44 | 0.23 | 2.67 | 0.12 | 1.98 | 0.11 | 2.68 | 0.12 |
| 8 | 1.60 | 0.08 | NA | NA | 1.69 | 0.09 | 1.52 | 0.07 |
| 9 | 1.15 | 0.05 | 1.14 | 0.05 | 1.25 | 0.07 | 1.06 | 0.05 |
| 10 | 1.62 | 0.07 | 1.63 | 0.09 | 1.74 | 0.11 | 1.51 | 0.09 |
| 11 | 2.75 | 0.07 | 2.69 | 0.16 | 2.90 | 0.16 | 2.67 | 0.15 |
| 12 | 1.21 | 0.10 | 1.12 | 0.09 | 1.42 | 0.09 | 1.10 | 0.08 |

**Table S6.** Among- vs. within-site variability in hourly inversion frequency and strength (ISLR). Within-site variability of each site is 1 SE of the mean across all 3 transects per site. Among-site variability is 1 SE of the mean across all 3 sites.

|  |  | Among-site variability | Within-site variability | | |
| --- | --- | --- | --- | --- | --- |
| Variable | Hourly interval |  | Shallow Basin | Medium Valley | Deep Valley |
| Hourly inversion frequency | 0 | 4.46 | 2.95 | 3.12 | 0.06 |
|  | 1 | 4.98 | 1.73 | 3.29 | 0.74 |
|  | 2 | 4.75 | 1.10 | 3.27 | 1.22 |
|  | 3 | 4.69 | 3.11 | 3.66 | 0.43 |
|  | 4 | 4.23 | 3.70 | 2.98 | 0.83 |
|  | 5 | 4.34 | 4.37 | 3.30 | 1.04 |
|  | 6 | 3.34 | 3.25 | 4.15 | 0.69 |
|  | 7 | 3.69 | 4.03 | 3.40 | 1.05 |
|  | 8 | 6.55 | 6.67 | 1.77 | 2.07 |
|  | 9 | 9.76 | 13.83 | 4.75 | 2.77 |
|  | 10 | 13.67 | 16.55 | 10.98 | 2.57 |
|  | 11 | 14.90 | 17.35 | 13.92 | 2.70 |
|  | 12 | 15.05 | 17.92 | 17.64 | 2.30 |
|  | 13 | 13.27 | 19.53 | 14.87 | 2.25 |
|  | 14 | 11.77 | 16.96 | 13.72 | 2.55 |
|  | 15 | 8.96 | 13.42 | 10.56 | 2.55 |
|  | 16 | 4.55 | 5.67 | 2.45 | 1.26 |
|  | 17 | 2.61 | 1.12 | 2.30 | 1.39 |
|  | 18 | 3.19 | 2.28 | 3.82 | 2.78 |
|  | 19 | 3.47 | 0.52 | 4.32 | 3.76 |
|  | 20 | 2.60 | 0.95 | 3.60 | 2.90 |
|  | 21 | 3.28 | 3.67 | 3.40 | 3.12 |
|  | 22 | 3.88 | 4.38 | 2.95 | 1.41 |
|  | 23 | 4.57 | 4.97 | 3.46 | 1.16 |
| Hourly inversion strength (ISLR) | 0 | 0.18 | 0.20 | 0.25 | 0.08 |
|  | 1 | 0.17 | 0.26 | 0.24 | 0.06 |
|  | 2 | 0.19 | 0.27 | 0.26 | 0.09 |
|  | 3 | 0.20 | 0.27 | 0.21 | 0.12 |
|  | 4 | 0.22 | 0.30 | 0.22 | 0.10 |
|  | 5 | 0.22 | 0.26 | 0.21 | 0.09 |
|  | 6 | 0.24 | 0.29 | 0.21 | 0.09 |
|  | 7 | 0.24 | 0.24 | 0.23 | 0.10 |
|  | 8 | 0.28 | 0.21 | 0.19 | 0.08 |
|  | 9 | 0.39 | 0.31 | 0.08 | 0.05 |
|  | 10 | 0.57 | 0.49 | 0.27 | 0.11 |
|  | 11 | 0.59 | 0.61 | 0.44 | 0.10 |
|  | 12 | 0.63 | 0.48 | 0.26 | 0.14 |
|  | 13 | 0.44 | 0.62 | 0.21 | 0.17 |
|  | 14 | 0.40 | 0.41 | 0.13 | 0.03 |
|  | 15 | 0.34 | 0.16 | 0.06 | 0.17 |
|  | 16 | 0.12 | 0.23 | 0.18 | 0.16 |
|  | 17 | 0.07 | 0.21 | 0.18 | 0.13 |
|  | 18 | 0.12 | 0.20 | 0.17 | 0.08 |
|  | 19 | 0.15 | 0.14 | 0.23 | 0.07 |
|  | 20 | 0.18 | 0.13 | 0.25 | 0.08 |
|  | 21 | 0.19 | 0.09 | 0.25 | 0.08 |
|  | 22 | 0.19 | 0.13 | 0.24 | 0.10 |
|  | 23 | 0.17 | 0.11 | 0.24 | 0.11 |

**Table S7.** Mean transect temperature difference during inversions (T_diff_) for each hourly interval at the site-level and transect-level. Temperature difference was calculated by subtracting the temperature of the first/lowest plot from that of the last/highest plot in a transect and was not standardized by elevation (i.e., this table shows temperature differences across entire transects that vary in elevation). We show each site-level mean ± 1 SE of 3 transects and each transect-level mean ± 1 SE across all dates over the full shared study period for each hourly interval.

| **Shallow Basin** | | | | | | | | |
| --- | --- | --- | --- | --- | --- | --- | --- | --- |
|  | Site-level | | SB-1 | | SB-2 | | SB-3 | |
| Hourly interval | Mean T_diff_ (°C) | SE | Mean T_diff_ (°C) | SE | Mean T_diff_ (°C) | SE | Mean T_diff_ (°C) | SE |
| 0 | 1.17 | 0.30 | 1.71 | 0.10 | 1.13 | 0.09 | 0.66 | 0.07 |
| 1 | 1.24 | 0.36 | 1.89 | 0.11 | 1.20 | 0.09 | 0.63 | 0.07 |
| 2 | 1.24 | 0.38 | 1.95 | 0.12 | 1.14 | 0.08 | 0.63 | 0.06 |
| 3 | 1.22 | 0.39 | 1.94 | 0.12 | 1.12 | 0.08 | 0.61 | 0.07 |
| 4 | 1.30 | 0.40 | 2.03 | 0.13 | 1.22 | 0.09 | 0.64 | 0.06 |
| 5 | 1.29 | 0.36 | 1.91 | 0.13 | 1.30 | 0.09 | 0.65 | 0.05 |
| 6 | 1.27 | 0.39 | 2.01 | 0.14 | 1.12 | 0.08 | 0.67 | 0.05 |
| 7 | 1.31 | 0.38 | 2.05 | 0.13 | 1.09 | 0.07 | 0.79 | 0.06 |
| 8 | 1.42 | 0.47 | 2.35 | 0.15 | 1.02 | 0.07 | 0.89 | 0.07 |
| 9 | 1.59 | 0.68 | 2.94 | 0.16 | 1.00 | 0.06 | 0.83 | 0.08 |
| 10 | 1.75 | 0.92 | 3.57 | 0.19 | 1.01 | 0.06 | 0.67 | 0.06 |
| 11 | 1.77 | 0.94 | 3.64 | 0.18 | 0.89 | 0.05 | 0.78 | 0.10 |
| 12 | 1.60 | 0.84 | 3.27 | 0.15 | 0.83 | 0.04 | 0.70 | 0.10 |
| 13 | 1.52 | 0.88 | 3.29 | 0.16 | 0.73 | 0.04 | 0.55 | 0.09 |
| 14 | 1.24 | 0.67 | 2.58 | 0.12 | 0.53 | 0.04 | 0.62 | 0.11 |
| 15 | 0.90 | 0.38 | 1.65 | 0.09 | 0.49 | 0.05 | 0.56 | 0.08 |
| 16 | 0.62 | 0.26 | 1.13 | 0.09 | 0.42 | 0.05 | 0.32 | 0.04 |
| 17 | 0.63 | 0.15 | 0.94 | 0.12 | 0.47 | 0.07 | 0.48 | 0.08 |
| 18 | 0.74 | 0.17 | 1.06 | 0.14 | 0.70 | 0.08 | 0.48 | 0.08 |
| 19 | 0.79 | 0.15 | 1.07 | 0.11 | 0.73 | 0.06 | 0.57 | 0.07 |
| 20 | 0.97 | 0.17 | 1.27 | 0.11 | 0.93 | 0.09 | 0.70 | 0.08 |
| 21 | 1.06 | 0.16 | 1.39 | 0.10 | 0.93 | 0.08 | 0.87 | 0.11 |
| 22 | 1.11 | 0.19 | 1.48 | 0.10 | 1.05 | 0.09 | 0.82 | 0.12 |
| 23 | 1.13 | 0.24 | 1.52 | 0.09 | 1.16 | 0.09 | 0.71 | 0.09 |
| **Medium Valley** | | | | | | | | |
|  | Site-level | | MV-1 | | MV-2 | | MV-3 | |
| Hourly interval | Mean T_diff_ (°C) | SE | Mean T_diff_ (°C) | SE | Mean T_diff_ (°C) | SE | Mean T_diff_ (°C) | SE |
| 0 | 2.44 | 0.36 | 2.14 | 0.21 | 2.02 | 0.19 | 3.16 | 0.25 |
| 1 | 2.39 | 0.36 | 2.05 | 0.21 | 2.00 | 0.18 | 3.11 | 0.23 |
| 2 | 2.58 | 0.37 | 2.34 | 0.22 | 2.10 | 0.19 | 3.31 | 0.24 |
| 3 | 2.63 | 0.29 | 2.42 | 0.23 | 2.28 | 0.20 | 3.20 | 0.24 |
| 4 | 2.75 | 0.30 | 2.45 | 0.23 | 2.45 | 0.21 | 3.35 | 0.26 |
| 5 | 2.71 | 0.27 | 2.55 | 0.26 | 2.33 | 0.23 | 3.24 | 0.26 |
| 6 | 2.80 | 0.24 | 2.71 | 0.28 | 2.43 | 0.25 | 3.26 | 0.27 |
| 7 | 2.92 | 0.29 | 2.80 | 0.32 | 2.50 | 0.28 | 3.46 | 0.31 |
| 8 | 3.00 | 0.23 | 2.91 | 0.30 | 2.65 | 0.28 | 3.43 | 0.31 |
| 9 | 2.76 | 0.11 | 2.90 | 0.30 | 2.54 | 0.27 | 2.85 | 0.32 |
| 10 | 2.53 | 0.19 | 2.90 | 0.24 | 2.38 | 0.23 | 2.29 | 0.27 |
| 11 | 2.01 | 0.45 | 2.91 | 0.21 | 1.51 | 0.13 | 1.62 | 0.21 |
| 12 | 1.65 | 0.31 | 2.26 | 0.15 | 1.32 | 0.13 | 1.36 | 0.18 |
| 13 | 1.81 | 0.37 | 2.54 | 0.20 | 1.35 | 0.13 | 1.56 | 0.29 |
| 14 | 1.30 | 0.12 | 1.49 | 0.13 | 1.08 | 0.15 | 1.34 | 0.23 |
| 15 | 1.38 | 0.12 | 1.16 | 0.10 | 1.55 | 0.19 | 1.42 | 0.23 |
| 16 | 1.24 | 0.36 | 0.73 | 0.12 | 1.05 | 0.17 | 1.93 | 0.28 |
| 17 | 1.51 | 0.39 | 0.89 | 0.18 | 1.39 | 0.23 | 2.24 | 0.28 |
| 18 | 1.60 | 0.28 | 1.29 | 0.20 | 1.35 | 0.20 | 2.17 | 0.26 |
| 19 | 1.74 | 0.33 | 1.41 | 0.18 | 1.40 | 0.19 | 2.40 | 0.23 |
| 20 | 1.96 | 0.36 | 1.52 | 0.19 | 1.69 | 0.16 | 2.67 | 0.22 |
| 21 | 2.10 | 0.34 | 1.71 | 0.21 | 1.80 | 0.19 | 2.77 | 0.24 |
| 22 | 2.28 | 0.37 | 1.91 | 0.22 | 1.92 | 0.20 | 3.01 | 0.24 |
| 23 | 2.34 | 0.36 | 2.00 | 0.20 | 1.95 | 0.20 | 3.06 | 0.24 |
| **Deep Valley** | | | | | | | | |
|  | Site-level | | DV-1 | | DV-2 | | DV-3 | |
| Hourly interval | Mean T_diff_ (°C) | SE | Mean T_diff_ (°C) | SE | Mean T_diff_ (°C) | SE | Mean T_diff_ (°C) | SE |
| 0 | 2.20 | 0.10 | 2.00 | 0.27 | 2.29 | 0.25 | 2.31 | 0.26 |
| 1 | 2.25 | 0.01 | 2.23 | 0.29 | 2.27 | 0.24 | 2.24 | 0.29 |
| 2 | 2.33 | 0.08 | 2.27 | 0.31 | 2.50 | 0.25 | 2.23 | 0.28 |
| 3 | 2.33 | 0.15 | 2.29 | 0.30 | 2.61 | 0.26 | 2.08 | 0.28 |
| 4 | 2.31 | 0.11 | 2.23 | 0.27 | 2.53 | 0.27 | 2.17 | 0.28 |
| 5 | 2.34 | 0.13 | 2.38 | 0.30 | 2.54 | 0.29 | 2.09 | 0.27 |
| 6 | 2.32 | 0.07 | 2.24 | 0.28 | 2.47 | 0.29 | 2.26 | 0.27 |
| 7 | 2.47 | 0.13 | 2.41 | 0.30 | 2.72 | 0.29 | 2.29 | 0.26 |
| 8 | 2.47 | 0.09 | 2.34 | 0.29 | 2.42 | 0.28 | 2.64 | 0.32 |
| 9 | 2.18 | 0.12 | 2.13 | 0.30 | 2.00 | 0.26 | 2.41 | 0.36 |
| 10 | 1.51 | 0.27 | 1.82 | 0.21 | 1.74 | 0.30 | 0.97 | 0.18 |
| 11 | 1.29 | 0.22 | 1.44 | 0.31 | 1.57 | 0.41 | 0.87 | 0.40 |
| 12 | 1.19 | 0.32 | 1.08 | 0.26 | 1.78 | 0.36 | 0.70 | 0.25 |
| 13 | 1.58 | 0.29 | 1.96 | 0.42 | 1.78 | 0.49 | 1.00 | NA^a^ |
| 14 | 1.43 | 0.12 | 1.38 | 0.36 | 1.25 | 0.38 | 1.65 | NA^a^ |
| 15 | 1.12 | 0.26 | 1.00 | 0.26 | 1.62 | 0.34 | 0.75 | 0.07 |
| 16 | 1.03 | 0.18 | 0.94 | 0.15 | 1.38 | 0.45 | 0.77 | 0.24 |
| 17 | 1.07 | 0.16 | 1.16 | 0.18 | 0.77 | 0.13 | 1.29 | 0.28 |
| 18 | 1.08 | 0.10 | 1.24 | 0.19 | 0.90 | 0.13 | 1.12 | 0.19 |
| 19 | 1.14 | 0.07 | 1.20 | 0.15 | 1.00 | 0.21 | 1.21 | 0.16 |
| 20 | 1.32 | 0.10 | 1.28 | 0.16 | 1.17 | 0.19 | 1.50 | 0.16 |
| 21 | 1.54 | 0.06 | 1.48 | 0.15 | 1.49 | 0.21 | 1.66 | 0.19 |
| 22 | 1.80 | 0.14 | 1.56 | 0.19 | 1.81 | 0.21 | 2.03 | 0.24 |
| 23 | 2.08 | 0.18 | 1.77 | 0.21 | 2.08 | 0.23 | 2.39 | 0.26 |

^a^Only 1 inversion occurred.
